# Stimuli-responsive nanocarriers for precision targeted and controlled antimicrobial drug delivery in drug-resistant infections

**DOI:** 10.3389/fmicb.2026.1803769

**Published:** 2026-06-12

**Authors:** Li Dai, Xiaoyan Sun

**Affiliations:** Clinical Laboratory, Danyang Hospital of Traditional Chinese Medicine, Jiangsu, China

**Keywords:** antimicrobial resistance, controlled drug release, infection microenvironment, stimuli-responsive nanocarriers, targeted antimicrobial delivery

## Abstract

Antimicrobial resistance (AMR) represents a critical and escalating threat to global health, driven not only by microbial evolution but also by fundamental limitations in the pharmacokinetic and spatial delivery of antimicrobial agents *in vivo*. Conventional antibiotics exhibit non-specific systemic distribution, frequently fail to achieve sustained therapeutic concentrations at infection sites, and expose both pathogenic and commensal microbiota to sub-inhibitory levels that accelerate resistance selection. In contrast, infected tissues exhibit distinct and dynamic microenvironmental features—including acidic pH, elevated reactive oxygen species, pathogen-associated enzymatic activity, hypoxia, and localized inflammatory signaling—that are largely absent in healthy tissues and provide exploitable triggers for targeted therapy. Stimuli-responsive nanocarriers are engineered to sense and respond to these pathological cues, enabling spatiotemporally controlled and infection-specific drug release while minimizing systemic exposure. In this review, we systematically analyze the mechanistic foundations of pH-, enzyme-, redox-, and multi-stimuli-responsive nanocarriers, with particular emphasis on how trigger-induced physicochemical transformations govern drug retention, activation, penetration, and release within drug-resistant infection niches. We further examine how these platforms address key resistance-associated barriers, including impaired tissue penetration, biofilm-associated tolerance, intracellular pathogen persistence, efflux-mediated drug extrusion, and enzymatic antibiotic degradation. Importantly, we provide a critical comparison between passive nanocarriers, stimuli-responsive systems, and free antibiotics, highlighting the conditions under which infection-synchronized delivery enhances antimicrobial efficacy, reduces off-target toxicity, and mitigates resistance-selective pressure. We also evaluate current translational challenges, including microenvironmental heterogeneity, trigger variability, long-term safety, scalable manufacturing, and regulatory complexity. Collectively, stimuli-responsive nanocarriers represent a paradigm shift from passive systemic exposure toward context-aware, site-selective antimicrobial intervention, offering a promising strategy to overcome persistent limitations in anti-infective therapy and address AMR beyond the pace of conventional antibiotic discovery.

## 1 Rationale and clinical need

Antimicrobial resistance has escalated into a global health crisis with profound clinical consequences. The 2022 Global Burden of Disease study estimated that AMR was directly responsible for 1.27 million deaths worldwide in 2019 and implicated in up to 5 million deaths when considering associated infections, ranking it among the leading causes of mortality globally (Murray et al., 2022). In bloodstream infections alone, AMR has been associated with a 58% increase in mortality, nearly doubling ICU admission rates and extending hospital stays by approximately 7 days, with direct medical costs increasing by ~$12,000 per case in low- and middle-income countries (Murray et al., 2022). Despite the availability of dozens of antibiotic classes, current antimicrobial therapies face intrinsic limitations that compromise clinical efficacy and fuel resistance development (David et al., 2018; Hassall et al., 2024). A key pharmacokinetic challenge is poor penetration into infected tissues and cellular compartments, particularly in sites characterized by physical and biological barriers such as biofilms and intracellular niches. Conventional systemic antibiotics often fail to achieve therapeutic concentrations at the precise loci of infection without generating toxicity in healthy tissues. Even antibiotics with high systemic bioavailability may be unable to achieve inhibitory concentrations within biofilms or within macrophages harbouring intracellular pathogens (Flemming et al., 2016; Pearson et al., 2017).

**Figure 1:**
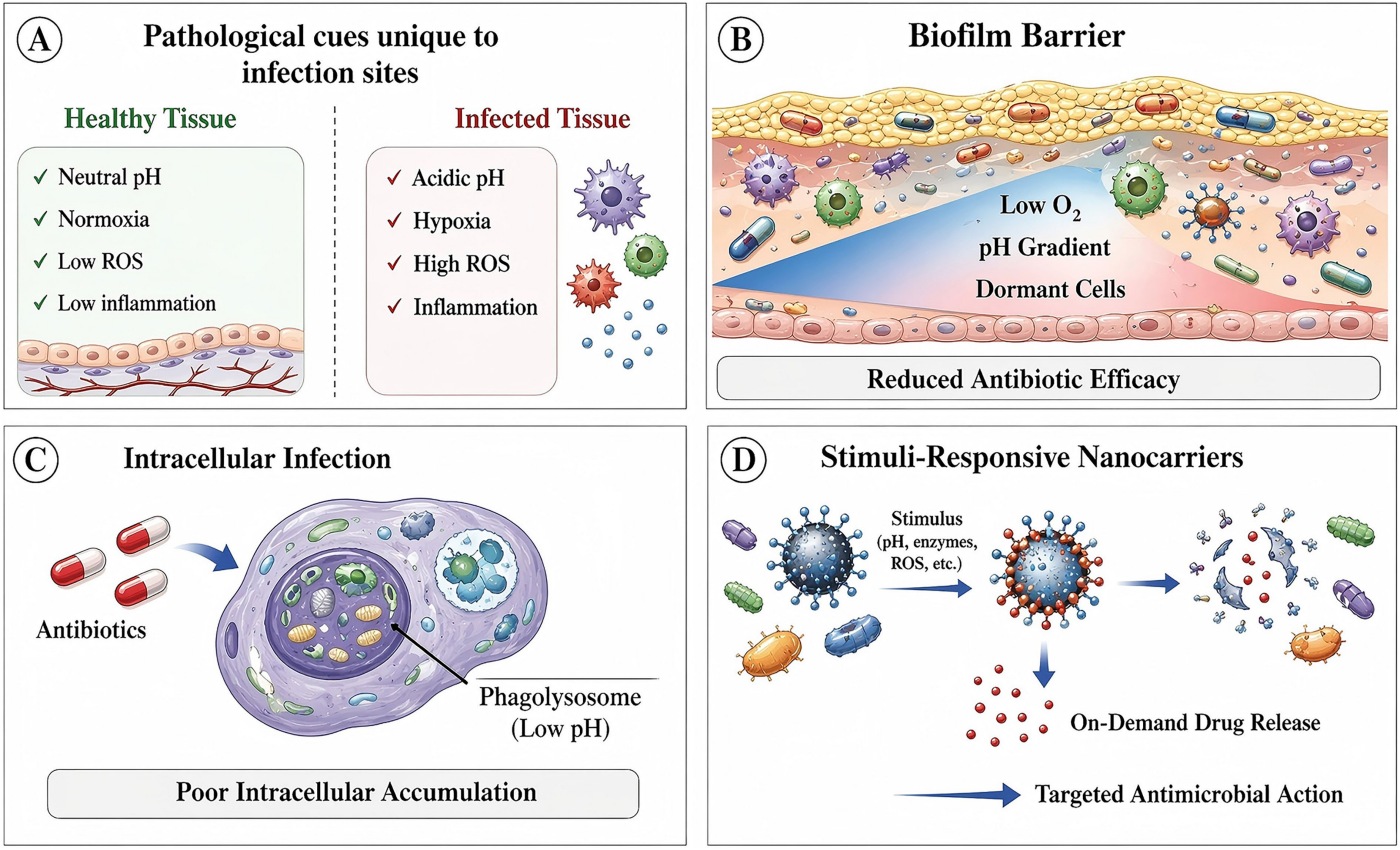
Key infection microenvironmental cues exploited for stimuli-responsive antimicrobial drug release **(A)** Pathological features of infected tissues. In contrast to healthy tissues, infection sites exhibit acidic pH, hypoxia, elevated reactive oxygen species (ROS), and inflammation, which can be harnessed as endogenous triggers for targeted antimicrobial delivery; **(B)** Biofilm-associated barriers. Biofilms generate heterogeneous microenvironments characterized by oxygen limitation, pH gradients, and metabolically dormant bacterial subpopulations, resulting in impaired antibiotic penetration and reduced therapeutic efficacy; **(C)** Intracellular bacterial infection. Intracellular pathogens residing within acidic phagolysosomal compartments limit antibiotic accumulation and activity, contributing to treatment failure and persistence; **(D)** Stimuli-responsive nanocarriers. Nanocarriers engineered to respond to infection-associated microenvironmental cues enable on-demand drug release and targeted antimicrobial action, improving efficacy against biofilm-associated and intracellular pathogens.

Biofilms, structured microbial communities embedded in an extracellular polymeric matrix, pose a paradigmatic example of microenvironmental barriers to therapy. The dense extracellular matrix limits drug diffusion, reduces local oxygen levels, creates pH gradients, and harbours slow-growing or metabolically dormant cells, all of which diminish antibiotic efficacy. These barriers necessitate high systemic doses to partially penetrate such microenvironments, increasing the risk of adverse events and perturbation of the host microbiota (Flemming et al., 2016; Hall and Mah, 2017). Sub-therapeutic exposure in which local drug concentrations at the site of infection fall below the minimal inhibitory concentration (MIC) has been shown to select for resistant subpopulations, accelerating the evolution of resistance even at concentrations previously considered adequate for clinical effect (Gullberg et al., 2011). In addition to penetration limitations, conventional antibiotic dosing typically results in fluctuating drug levels, with peaks and troughs that can overshoot toxicity thresholds or fall into sub-inhibitory ranges. These dynamics not only compromise antibacterial activity but also disrupt commensal microbiota and contribute to collateral host tissue damage, chronic inflammation, and dysbiosis. Systemic toxicity remains a tangible clinical problem; for instance, aminoglycosides are limited by nephrotoxicity and ototoxicity at doses required for difficult infections, and fluoroquinolones have been associated with tendon rupture and central nervous system effects (Blaser, 2016; Morales et al., 2019). Given these challenges, there is a compelling rationale for therapies that can precisely deliver antimicrobial agents to infection sites and sustain effective local exposure while minimizing off-target systemic exposure. The concept of drug delivery tailored to the unique microenvironment of infected tissues has gained traction as a strategy to overcome pharmacokinetic barriers and reduce resistance selection pressure. These pathological microenvironments are characterized by features distinct from healthy tissues, including acidic pH, altered redox balance, elevated concentrations of bacterial enzymes, hypoxia, and localized inflammatory processes. Such cues offer exploitable triggers for on-demand drug release (Figure 1). Stimuli-responsive nanocarriers are engineered drug delivery systems that undergo site-specific structural transformations in response to endogenous or exogenous triggers, enabling controlled release of payloads exactly when and where needed (Mura et al., 2013; Zhou et al., 2018). These triggers can be intrinsic to the infection microenvironment (low pH, high enzyme activity, oxidative stress) or applied externally (light, temperature, ultrasound) to spatially and temporally control drug liberation (Torchilin, 2014). By synchronizing antimicrobial release with pathological conditions, stimuli-responsive nanocarriers enhance localized drug concentrations while reducing systemic exposure and collateral effects. pH-responsive systems exploit the acidic milieu of infection sites often several pH units lower than normal physiological pH to destabilize carrier structures or cleave pH-labile linkages, releasing cargo specifically in infected tissue (Gao et al., 2010; Mura et al., 2013). Enzyme-responsive carriers can leverage overexpression of bacterial proteases or β-lactamases in infected environments to trigger antimicrobial release only in the presence of pathogens. Redox-responsive systems take advantage of elevated reactive oxygen species (ROS) and altered redox states in inflamed tissues to initiate drug release through cleavable disulfide or thioketal bonds (Gao and Xiong, 2021). The stimuli-responsive delivery platforms address multiple pharmacokinetic and pharmacodynamic shortcomings of traditional antibiotics by achieving spatiotemporal control, enhanced tissue and cellular targeting, and reduction of off-target distribution (Zhou et al., 2018). Such precision delivery not only amplifies therapeutic efficacy against drug-resistant pathogens but also minimizes the evolutionary pressure that drives AMR. By aligning drug release mechanisms with pathological cues rather than systemic distribution alone, these nanocarriers represent a promising therapeutic emerging strategy from empirical, systemic antibiotic administration to infection-context-aware, precision antimicrobial therapy.

## 2 Infection microenvironment as a trigger source

A defining limitation of conventional antimicrobial therapy is its failure to account for the profound physicochemical divergence between infected and healthy tissues (Table 1). Infection sites are not passive targets; rather, they represent dynamic pathological microenvironments shaped by microbial metabolism, host immune responses, and tissue damage. These alterations generate endogenous cues largely absent from healthy tissues that can be rationally exploited as triggers for controlled antimicrobial release. Importantly, these triggers are spatially confined and temporally linked to disease activity, providing an intrinsic mechanism for selective drug activation (Liu et al., 2024a; Nnenna Ugwu et al., 2025). A critical translational consideration is that the infection microenvironment is highly heterogeneous rather than biologically uniform. The magnitude, duration, and spatial distribution of pathological cues such as acidity, oxidative stress, bacterial enzyme secretion, hypoxia, and inflammatory mediator release vary substantially between patients, pathogen species, anatomical compartments, and stages of disease progression (Bjarnsholt et al., 2022). For example, acute abscesses may exhibit pronounced acidosis and neutrophil-driven ROS bursts, whereas chronic biofilm infections often display steep micron-scale gradients in oxygen, nutrient availability, and pH together with metabolically dormant bacterial subpopulations. Likewise, host factors including diabetes, vascular insufficiency, immunosuppression, renal dysfunction, age, and prior antibiotic exposure can significantly reshape tissue perfusion, immune activation, and local trigger intensity. This heterogeneity has direct consequences for infection-responsive nanocarriers: a trigger robust in one setting may be weak, transient, or spatially restricted in another, leading to incomplete activation, delayed release, burst release, or variable therapeutic efficacy. Consequently, future platform design should prioritize adaptable threshold tuning, multi-stimuli logic gating, patient-stratified deployment, and integration with diagnostic biomarkers capable of quantifying the local infection state before or during treatment.

**Table 1:** Comparative features of healthy vs. infected tissue microenvironments.

Parameter	Healthy tissue	Infected tissue
pH (Flemming et al., 2016)	7.35–7.45	5.0–6.5
ROS levels (Nathan and Cunningham-Bussel, 2013)	Basal (<10^−9^ M)	Elevated (10^−7^–10^−6^ M)
Bacterial enzymes (Kunjachan et al., 2013)	Absent	High local activity
Oxygen tension (Flemming et al., 2016)	40–60 mmHg	<10 mmHg
Inflammatory mediators (Dinarello, 2000)	Low	High (localized)
Drug penetration (Pearson et al., 2017)	Uniform	Restricted, heterogeneous

### 2.1 Acidic pH

Localized acidosis is one of the most consistent hallmarks of bacterial infection. In healthy tissues, extracellular pH is tightly regulated around 7.35–7.45, whereas infected sites commonly exhibit pH values ranging from 6.5 to as low as 5.0 as a consequence of anaerobic glycolysis, lactate accumulation, and impaired perfusion (Ding et al., 2022). Abscesses and chronic wound infections frequently display pH values of 5.5–6.2, reflecting sustained hypoxia and metabolic stress within infected tissue compartments (Percival et al., 2014). Biofilms further exacerbate local pH heterogeneity, with microdomains reaching pH values below 5.5 due to restricted diffusion, accumulation of acidic metabolites, and metabolic stratification within the extracellular polymeric matrix (Price and Skaar, 2025). Intracellular pathogens residing within phagolysosomes are exposed to even more acidic environments, with vacuolar pH values typically ranging from 4.5 to 5.0 during host immune responses (Lee et al., 2020). These pronounced pH differentials provide a robust and reliable trigger for nanocarriers incorporating acid-labile linkers, protonatable polymers, or charge-reversal mechanisms, enabling selective destabilization and drug release at infected sites (Huang and Brazel, 2001; Mura et al., 2013). Recent quantitative imaging and infection-model studies have demonstrated that pH-responsive nanoparticles can enhance local antibiotic such as vancomycin concentrations by significate higher fold in acidic infection microenvironments compared with non-responsive formulations, without increasing systemic drug exposure (Kolge et al., 2026).

### 2.2 Elevated reactive oxygen species (ROS)

Infection-associated inflammation leads to marked oxidative stress driven primarily by activated neutrophils and macrophages at the site of infection. While basal ROS levels in healthy tissues are tightly regulated and typically remain below 10^−9^ M hydrogen peroxide equivalents, infected and inflamed tissues exhibit ROS concentrations that are one to two orders of magnitude higher, particularly during acute bacterial infections and sepsis (Nathan and Cunningham-Bussel, 2013; Winterbourn and Hampton, 2015). Activated phagocytes generate superoxide anions, hydrogen peroxide, and hydroxyl radicals through NADPH oxidase (NOX2) and myeloperoxidase (MPO)–dependent pathways, establishing a highly oxidative local microenvironment essential for microbial killing but detrimental to surrounding tissue (Segal, 2008; Flannagan et al., 2012). This redox imbalance has been quantitatively documented in murine and ex-vivo infection models, where ROS concentrations at inflammatory foci commonly reach 10^−7^–10^−6^ M, levels sufficient to cleave oxidation-sensitive chemical moieties such as thioketal, boronic ester, and thioether bonds incorporated into redox-responsive delivery systems (Liang and Liu, 2016). Recent imaging and biosensor-based studies further confirm that these elevated ROS levels are spatially confined to infected tissues and correlate with disease severity and immune activation (Abdesselem et al., 2023). ROS-responsive nanocarriers exploit this pathological redox disparity to achieve inflammation-localized drug release, enabling selective payload liberation in infected tissues while limiting systemic exposure. Such systems have demonstrated particular relevance in chronic lung infections, implant-associated infections, and biofilm-mediated inflammatory environments, where sustained oxidative stress persists despite conventional antibiotic therapy (Liu et al., 2024b).

### 2.3 Bacterial enzymes

Unlike host tissues, infected microenvironments are enriched with pathogen-derived enzymes that directly reflect bacterial burden and metabolic activity. β-lactamases, proteases, phospholipases, and lipases are frequently secreted at high local concentrations, particularly by multidrug-resistant pathogens, and are largely absent from healthy tissues (Tooke et al., 2019; Bush and Bradford, 2020). The β-lactamase activity in resistant Enterobacterales infections can reach levels sufficient to hydrolyze conventional β-lactam antibiotics within minutes, severely compromising therapeutic efficacy (Tooke et al., 2019; Gach et al., 2024). Similarly, extracellular protease production in *Staphylococcus aureus* biofilms has been shown to be several-fold higher than in planktonic cultures, contributing to tissue invasion, immune evasion, and antibiotic tolerance (Lister and Horswill, 2014). Enzyme-responsive nanocarriers convert this pathogenic liability into a therapeutic targeting advantage by incorporating enzyme-cleavable linkers such as β-lactam, peptide, or ester bonds thereby enabling antimicrobial release exclusively in the presence of active bacterial enzymes (Mura et al., 2013). Experimental studies have demonstrated that β-lactamase-responsive delivery systems can selectively activate antibiotics at infected sites while remaining inert in sterile inflammatory tissues, resulting in enhanced bacterial killing and significantly improved therapeutic indices in-vivo (Nkune and Abrahamse, 2025).

### 2.4 Hypoxia and toxin secretion

Impaired perfusion, vascular damage, and elevated metabolic demand render many infection sites profoundly hypoxic, with local oxygen tensions frequently falling below 10 mmHg, compared with 40–60 mmHg in healthy tissues (Hall and Mah, 2017) particularly pronounced in abscesses, biofilms, necrotic tissue cores, and chronic wound infections, where restricted diffusion and high bacterial respiration exacerbate oxygen depletion (Lewis, 2010). Experimental measurements in murine and human infection models have confirmed sustained hypoxia as a defining feature of deep-seated and biofilm-associated infections. Concurrently, many bacterial pathogens secrete toxins, including pore-forming toxins (*α*-hemolysin) and secreted exotoxins, which further disrupt tissue integrity, impair cellular homeostasis, and aggravate local ischemia and inflammation (Jiang et al., 2023). These toxins synergize with hypoxia to promote immune evasion and antibiotic tolerance, reinforcing the pathological microenvironment characteristic of chronic infections (Ulhuq and Mariano, 2022). Hypoxia-responsive nanocarriers often incorporating hypoxia-activated prodrugs, nitroimidazole-based moieties, or redox-sensitive linkers exploit these oxygen gradients to enable selective drug release within oxygen-deprived infected tissues (Mamnoon et al., 2020). Although less extensively explored than pH- or enzyme-responsive systems, hypoxia-responsive delivery platforms show particular promise for deep-seated, biofilm-mediated, and chronic infections, where oxygen deprivation is sustained and conventional antibiotic penetration is limited (Hall and Mah, 2017).

### 2.5 Host inflammatory mediators

Infected tissues are characterized by elevated concentrations of host-derived inflammatory mediators including pro-inflammatory cytokines such as tumor necrosis factor-α (TNF-α), interleukin-1β (IL-1β), IL-6 and chemokines, which are secreted by activated macrophages, neutrophils, and other immune cells in response to pathogen presence and tissue injury (Dinarello, 2000). These cytokines and matrix-degrading enzymes not only remodel the extracellular matrix but also enhance local vascular permeability through endothelial activation and leukocyte recruitment, thereby promoting the extravasation and retention of nanoparticles at inflamed sites an effect analogous to the enhanced permeability and retention (EPR) phenomenon described in inflammatory settings (Maeda et al., 2016). Importantly, circulating and tissue levels of inflammatory mediators correlate closely with disease severity in infectious and inflammatory conditions, reflecting ongoing immune activation and tissue damage (Tracey and Cerami, 1994; Hunter and Jones, 2015). Elevated levels of TNF-α, IL-1β, and IL-6 are consistently detectable in severe bacterial infections, chronic non-healing wounds, and inflammatory lung diseases, and have been widely proposed as biomarkers of active and ongoing inflammation (Tracey and Cerami, 1994; Dinarello, 2000; Schulte et al., 2013). These mediators directly promote endothelial activation, intercellular gap formation, and leukocyte recruitment, thereby increasing vascular permeability and facilitating preferential nanoparticle extravasation and retention within inflamed tissues compared with healthy counterparts (Tracey and Cerami, 1994; Fang et al., 2011). Because the infection microenvironment constitutes a multi-parameter trigger landscape characterized by elevated cytokines, protease activity, and altered vascular physiology, stimuli-responsive nanocarriers can be rationally engineered to integrate these cues such as inflammation-cleavable linkers or surface ligands targeting inflammatory receptors to achieve pathology-synchronized and site-specific antimicrobial release (Mura et al., 2013; Peng et al., 2021). By aligning therapeutic activation with disease-specific mediator profiles, these platforms can leverage host inflammatory responses to improve localization and transform pathological heterogeneity from a barrier into a targeting advantage for enhanced delivery and efficacy.

## 3 Classification of stimuli-responsive nanocarriers

**Figure 2:**
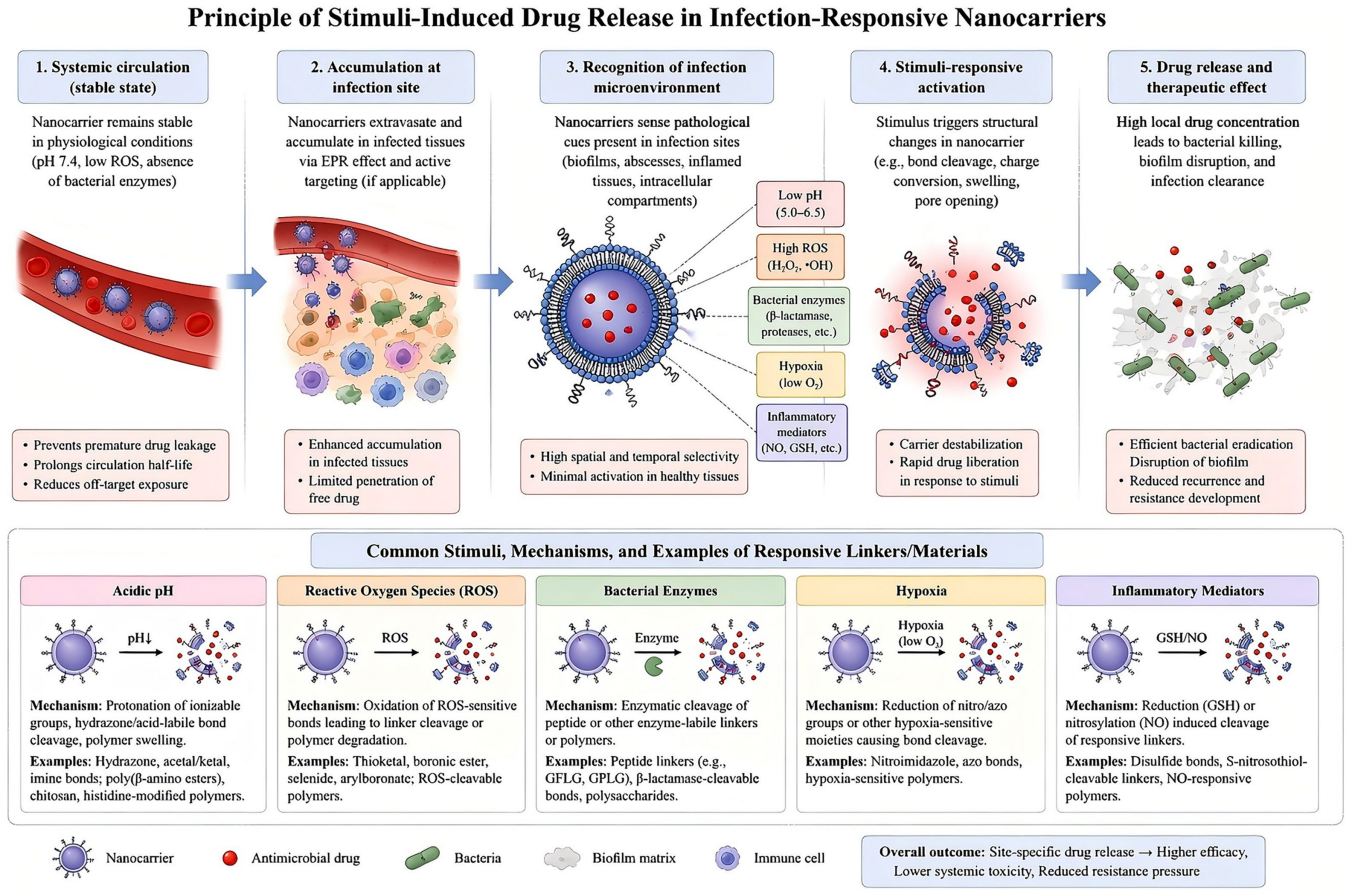
Principle of stimuli-induced drug release in infection-responsive nanocarriers. Stimuli-responsive nanocarriers remain stable during systemic circulation under physiological conditions, thereby minimizing premature drug leakage and off-target exposure. Following preferential accumulation at infected tissues, the carriers detect pathological microenvironmental cues including acidic pH, elevated reactive oxygen species (ROS), bacterial enzymes, hypoxia, and inflammatory mediators. These triggers induce structural or chemical transformations such as polymer swelling, linker cleavage, pore opening, charge conversion, or matrix destabilization, leading to localized antimicrobial release. Site-specific liberation of the payload enhances intralesional drug concentration, improves bacterial killing and biofilm disruption, facilitates clearance of intracellular pathogens, and reduces systemic toxicity and resistance-selective pressure compared with non-responsive delivery systems.

**Figure 3:**
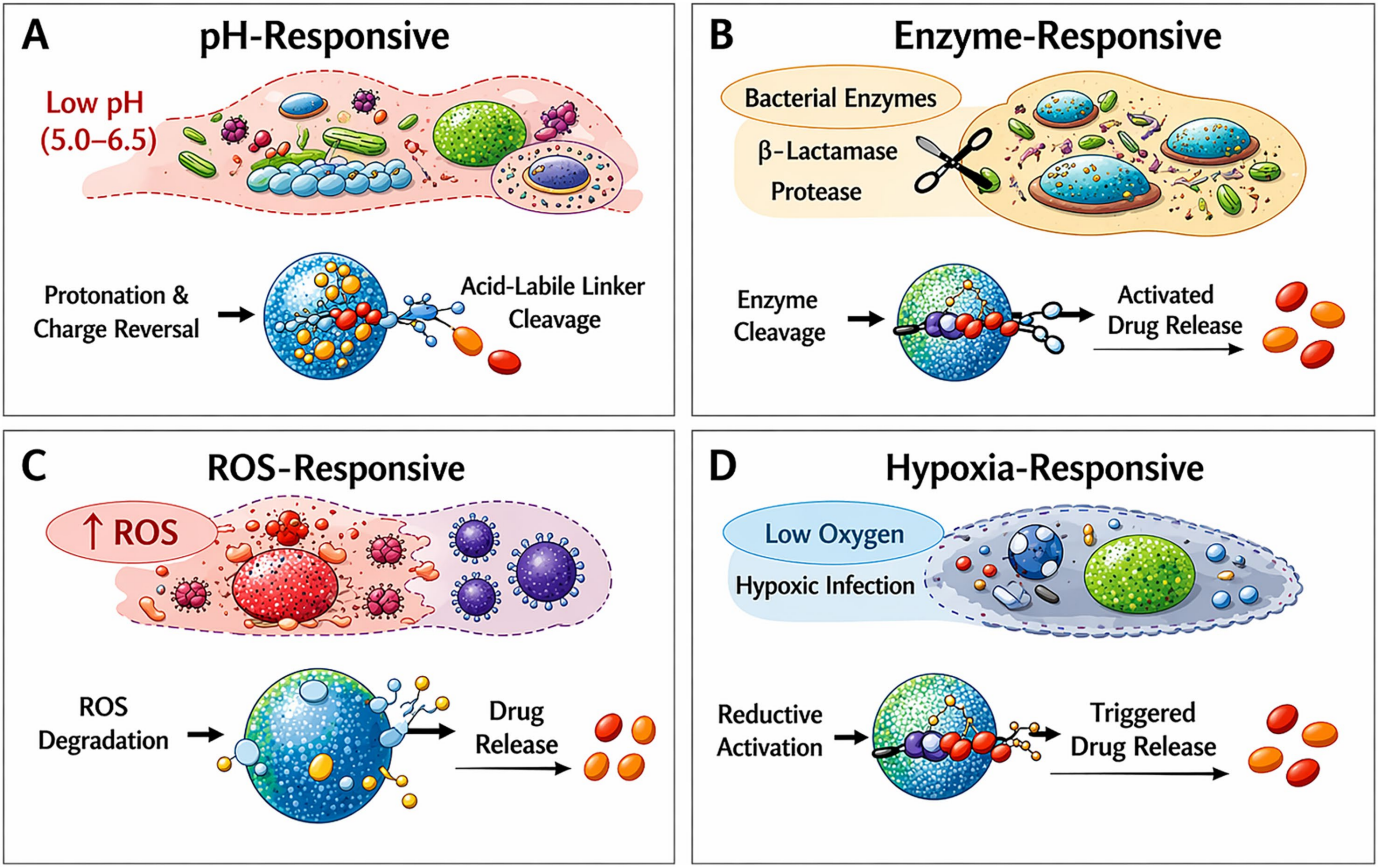
Classification and mechanisms of stimuli-responsive nanocarriers for infection-targeted antimicrobial delivery **(A)** pH-responsive systems. Acidic microenvironments at infection sites (pH 5.0–6.5) induce protonation, charge reversal, and cleavage of acid-labile linkers, enabling site-specific drug release; **(B)** Enzyme-responsive systems. Bacterial enzymes, such as β-lactamases and proteases, selectively cleave carrier structures or linkers, activating antimicrobial drug release in pathogen-rich environments; **(C)** ROS-responsive systems. Elevated reactive oxygen species (ROS) levels at infected or inflamed sites trigger oxidative degradation of nanocarriers, resulting in controlled drug release; **(D)** Hypoxia-responsive systems. Low-oxygen conditions characteristic of chronic and biofilm-associated infections activate reductive moieties within nanocarriers, leading to hypoxia-triggered antimicrobial release.

Stimuli-responsive nanocarriers are broadly classified according to the dominant pathological cue that governs their structural transformation and drug-release behavior (Figure 2) (Mura et al., 2013). Among these, pH-responsive nanocarriers represent the most extensively investigated and comparatively mature subclass, owing to the reproducible acidity of infection sites and the relative simplicity and robustness of pH-sensitive material design (Huang and Brazel, 2001; Mura et al., 2013). Acidic microenvironments are consistently observed in biofilms, abscesses, and intracellular infection niches, making pH a reliable endogenous trigger across diverse infectious contexts (Hall and Mah, 2017). Consistent experimental evidence indicates that pH-responsive systems achieve improved targeting efficiency and enhanced antimicrobial activity compared with non-responsive carriers, particularly in biofilm-associated infections, poorly vascularized abscesses, and intracellular bacterial reservoirs, where conventional antibiotics exhibit limited penetration and sub-therapeutic exposure (Table 2) (Deiss-Yehiely et al., 2023). In multiple in-vitro and in-vivo infection models, pH-responsive nanocarriers have demonstrated superior bacterial killing and reduced systemic toxicity relative to free drugs or non-responsive formulations, supporting their translational relevance (Gui et al., 2023). Mechanistically, pH-responsive nanocarriers undergo proton-mediated physicochemical transitions, including polymer protonation, surface charge reversal, cleavage of acid-labile linkers, or destabilization of carrier architecture. These processes collectively enable triggered drug release, enhanced cellular uptake, and improved penetration into biofilms or intracellular compartments (Figure 3) (Huang and Brazel, 2001). By synchronizing antimicrobial release with infection-associated acidity, pH-responsive nanocarriers provide a rational strategy to enhance therapeutic index while minimizing off-target toxicity and reducing selective pressure for antimicrobial resistance (Farah et al., 2025).

**Table 2:** Classification of stimuli-responsive nanocarriers for infection-targeted antimicrobial delivery.

Stimulus type	Pathological trigger at infection site	Representative nanocarrier strategy	Mechanism of triggered release	Representative examples
pH-responsive (Mura et al., 2013)	Acidic microenvironment (pH 5.0–6.5 in biofilms, abscesses, intracellular compartments)	Acid-labile polymers, charge-reversal nanoparticles, protonatable micelles	Protonation, surface charge reversal, cleavage of acid-labile bonds, carrier destabilization	pH-responsive polymeric micelles for intracellular bacteria; charge-switching nanoparticles for biofilm penetration
Enzyme-responsive (Deiss-Yehiely et al., 2023)	Bacterial enzymes (β-lactamases, proteases, lipases) enriched at infection sites	Enzyme-cleavable linkers (β-lactam, peptide, ester bonds)	Enzymatic cleavage activates drug release only in presence of pathogens	β-lactamase-responsive antibiotic nanocarriers; protease-activated antimicrobial systems
ROS-responsive (Nathan and Cunningham-Bussel, 2013)	Elevated reactive oxygen species generated by activated phagocytes	ROS-cleavable polymers (thioketal, boronic ester linkages)	Oxidative cleavage of ROS-sensitive bonds triggers release	Thioketal-based nanoparticles releasing antibiotics under inflammatory ROS
Hypoxia-responsive (Hall and Mah, 2017)	Oxygen-deprived niches in biofilms, abscesses, necrotic tissue	Hypoxia-activated prodrugs, redox-sensitive carriers	Reduction-mediated activation or carrier destabilization under low oxygen	Hypoxia-responsive nanocarriers for deep-seated and chronic infections

### 3.1 pH-responsive nanocarriers

#### 3.1.1 Mechanistic principles

pH-responsive nanocarriers are engineered to remain structurally stable at physiological pH (~7.4) while undergoing predictable and rapid transformations under acidic conditions typical of infected tissues. Three dominant mechanistic strategies are employed. Protonation-induced polymer swelling is commonly achieved using polymers containing weakly basic functional groups (tertiary amines) (Liu et al., 2013; Deirram et al., 2019). Upon exposure to acidic environments (pH ≤ 6.5), protonation increases electrostatic repulsion within the polymer matrix, resulting in volumetric swelling and enhanced permeability. Quantitative release studies consistently demonstrate strong stimulus selectivity. pH-responsive polymeric nanoparticles typically release 60–85% of encapsulated antibiotic within 12–24 h at pH 5.5, whereas release at physiological pH 7.4 remains below 15–20% over the same interval. Hydrazone-linked systems exhibit cleavage half-lives of 2–8 h at pH ≤ 6.0, compared with >72 h at pH 7.4, indicating marked on/off release discrimination. Similarly, charge-switchable formulations show accelerated payload liberation after protonation-mediated destabilization in acidic biofilm environments (Han et al., 2022). Swelling-mediated release also increases drug diffusion coefficients by 3–6-fold under acidic conditions, facilitating rapid local drug accumulation. A second strategy involves acid-labile chemical bonds, including hydrazone, acetal, and imine linkages, which undergo cleavage in acidic environments (Du et al., 2010). These bonds exhibit half-lives of >72 h at pH 7.4 but are rapidly hydrolyzed at pH ≤ 6.0, with reported cleavage half-times ranging from 2 to 8 h, depending on linker chemistry (Bae et al., 2007). This sharp kinetic contrast enables minimal premature leakage during systemic circulation while ensuring efficient payload liberation at infection sites. Importantly, acid-labile systems have demonstrated significantly increased in local antibiotic concentration within infected tissues compared with free drug administration in animal infection models (Blanco et al., 2015). Charge-reversal mechanisms represent a third, increasingly sophisticated design principle (Gillies and Fréchet, 2005). These nanocarriers are engineered to possess a neutral or negatively charged surface at physiological pH to reduce nonspecific protein adsorption and prolong circulation time. Under acidic conditions, protonation or cleavage of shielding groups induces a transition to a positively charged surface (typically shifting *ζ*-potential from ~ − 10 mV to +15–25 mV). This charge conversion enhances electrostatic interactions with negatively charged bacterial membranes and biofilm matrices, resulting in significantly higher bacterial binding efficiency and significantly improved intracellular uptake by infected macrophages (Lebeaux et al., 2014). These mechanisms often combined within a single platform enable precise, pH-gated drug release, where acid-triggered nanocarrier destabilization is coupled to localized antimicrobial action.

#### 3.1.2 Targeted applications

The clinical relevance of pH-responsive nanocarriers is most evident in infection settings characterized by sustained acidosis. In abscesses, where extracellular pH commonly ranges from 5.5 to 6.2, pH-responsive systems achieve prolonged drug retention and reduced systemic exposure (Nekoofar et al., 2009). In-vivo studies report 2–3-fold higher intralesional antibiotic concentrations and a 40–60% reduction in bacterial burden compared with equivalent doses of free antibiotics (Hall and Mah, 2017). For intracellular pathogens, such as *Mycobacterium tuberculosis* and *Salmonella enterica*, pH-responsive nanocarriers exploit the acidic phagolysosomal environment (pH ~ 4.5–5.0). Quantitative uptake studies demonstrate that pH-triggered systems increase intracellular antibiotic accumulation to non-responsive nanoparticles, translating into significantly enhanced intracellular killing without increasing host-cell toxicity (Nekoofar et al., 2009; Flannagan et al., 2012). In biofilm-associated infections, pH gradients within the extracellular polymeric substance create localized acidic niches that activate pH-responsive release. Quantitative imaging studies show that charge-reversal pH-responsive nanoparticles penetrate 30–50% deeper into mature biofilms than non-responsive controls and maintain intrabiofilm antibiotic concentrations above MBEC for ≥24 h. In parallel, bacterial surface association assays report little higher binding efficiency after acid-triggered positive charge conversion (Li et al., 2024). The pH-responsive nanocarriers convert infection-associated acidosis into a quantitative release switch, enabling large on/off differences in antimicrobial exposure between healthy and infected tissues. Their mechanistic simplicity, robust performance metrics, and demonstrated efficacy across abscess, intracellular, and biofilm infections position them as a foundational platform within stimuli-responsive antimicrobial nanomedicine (Karimi et al., 2016).

### 3.2 Enzyme-responsive nanocarriers

Enzyme-responsive nanocarriers constitute a distinct and highly selective class of stimuli-responsive systems in which antimicrobial release is governed by pathogen-derived enzymatic activity rather than host-associated physicochemical changes (Xiong et al., 2012). Unlike pH-responsive platforms that exploit broadly shared infection characteristics, enzyme-responsive systems operate on the principle of biochemical specificity, enabling drug activation only in the presence of viable, metabolically active pathogens. These nanocarriers exhibit the highest trigger specificity among endogenous stimuli-responsive systems, making them particularly attractive for minimizing off-target drug release and collateral host toxicity (Table 3).

**Table 3:** Classification of enzyme-responsive nanocarriers for infection-targeted antimicrobial delivery.

Enzyme trigger	Major producing pathogens	Nanocarrier design strategy	Trigger mechanism	Representative validated examples
β-Lactamases (Bush and Bradford, 2020)	*Enterobacterales*, *Pseudomonas aeruginosa*, *Acinetobacter baumannii*	β-lactam or cephalosporin-capped nanoparticles; β-lactamase-cleavable prodrugs	Enzymatic hydrolysis of β-lactam moiety activates drug release	β-lactamase-responsive nanogels selectively releasing antibiotics in resistant bacteria
Bacterial proteases (Lister and Horswill, 2014)	*Staphylococcus aureus*,	Peptide-linked nanocarriers with protease-cleavable sequences	Proteolytic cleavage destabilizes carrier or liberates drug	Protease-activated antimicrobial delivery systems targeting biofilm infections
Esterases/lipases (Hosseini et al., 2022)	*Mycobacterium tuberculosis*, *Staphylococcus aureus*	Ester-linked prodrugs or lipid-based nanocarriers	Enzymatic ester hydrolysis triggers drug activation	Esterase-responsive antibiotic prodrugs for intracellular bacteria
Phospholipases (Ahmad-Mansour et al., 2021)	*Clostridium* spp., *Listeria monocytogenes*	Phospholipid-based carriers sensitive to bacterial phospholipases	Membrane degradation releases payload	Phospholipase-triggered antimicrobial release
General bacterial enzymes (de la Rica et al., 2012)	Multiple MDR pathogens	Enzyme-responsive polymeric nanogels	Enzyme-mediated cleavage of linker polymers	Broad-spectrum enzyme-responsive nanogels

#### 3.2.1 Mechanistic principles

The dominant mechanism underlying enzyme-responsive nanocarriers is the cleavage of peptide or ester linkers that tether antimicrobial payloads to the carrier matrix or stabilize the nanostructure. These linkers are designed to remain stable under physiological conditions but undergo rapid degradation in the presence of enzymes overexpressed or secreted by pathogenic bacteria, including proteases, lipases, and β-lactamases, enabling site-specific drug release (Mura et al., 2013; Tooke et al., 2019). Peptide linkers responsive to bacterial proteases are among the most widely used designs. Proteases such as aureolysin, elastase, gelatinase, and V8 protease are secreted at high local concentrations by clinically relevant pathogens including *Staphylococcus aureus*, *Pseudomonas aeruginosa*, and *Enterococcus faecalis*. Kinetic studies demonstrate that protease-cleavable peptide linkers exhibit >80–90% structural integrity over 48–72 h in enzyme-free physiological media, while undergoing rapid cleavage within 1–6 h in the presence of bacterial protease concentrations representative of infected tissues (Deng et al., 2017). This sharp kinetic contrast results in 10- to 50-fold differences in drug release rates between infected and non-infected environments (Hu et al., 2014). Ester-based linkers provide an alternative strategy, particularly for pathogens that secrete phospholipases or esterases. These linkers can undergo selective hydrolysis in the presence of bacterial enzymes while remaining relatively stable under physiological conditions when appropriately shielded within nanocarrier architectures (de la Rica et al., 2012; Mura et al., 2013). Quantitative release experiments have shown that ester-responsive systems can achieve 70–85% drug release within 24 h in enzyme-rich infection models, compared with <10–15% release in sterile inflammatory conditions. A particularly powerful subclass of enzyme-responsive systems exploits pathogen-specific enzymatic activation, most notably β-lactamase-responsive nanocarriers (Bush and Bradford, 2020). β-lactamase activity is a defining feature of many drug-resistant Gram-negative and Gram-positive bacteria and is largely absent in mammalian tissues. Nanocarriers incorporating β-lactamase-cleavable motifs convert resistance enzymes traditionally viewed as therapeutic liabilities into targeting triggers. In resistant infection models, such systems have demonstrated >5-fold increases in intralesional antibiotic concentration and 50–70% reductions in bacterial load relative to free antibiotic administration, despite identical systemic dosing (Tooke et al., 2019). These mechanisms ensure that enzymatic activity functions not merely as a release cue but as a biological verification signal, confirming the presence of pathogenic bacteria before drug activation occurs.

#### 3.2.2 Advantages over other stimuli-responsive systems

A principal advantage of enzyme-responsive nanocarriers is their exceptionally high pathogen specificity. Because enzymatic triggers are directly linked to bacterial metabolism and virulence, drug release is inherently coupled to active infection, reducing the likelihood of premature activation in non-infected acidic or inflamed tissues (Hu et al., 2014). Comparative biodistribution studies indicate that enzyme-responsive systems exhibit 30–60% lower off-target drug release in healthy organs compared with pH-responsive counterparts, particularly in organs prone to physiological acidosis or inflammation (Hofer, 2021). Another critical advantage is the reduction of off-target drug exposure, which has direct implications for antimicrobial resistance. By confining drug release to enzyme-rich infection sites, these systems minimize sub-inhibitory antibiotic exposure in commensal microbiota. In longitudinal animal studies, enzyme responsive delivery has been associated with significantly reduced emergence of resistant subpopulations, with resistance frequencies decreasing by approximately 1–2 orders of magnitude compared with free antibiotic treatment (Hu et al., 2014). From a pharmacodynamic perspective, enzyme-responsive nanocarriers enable self-amplifying release behavior: higher bacterial burden results in greater enzymatic activity, which in turn accelerates drug release. This feedback mechanism aligns antimicrobial exposure with infection severity, a feature not achievable with conventional dosing or passive nanocarriers.

#### 3.2.3 Clinical and translational relevance

Enzyme-responsive nanocarriers are particularly well suited for drug-resistant infections, where enzymatic activity is often upregulated as part of resistance mechanisms. In biofilm-associated infections, extracellular enzyme accumulation within the biofilm matrix enables localized drug activation even when diffusion is limited (Flemming and Wingender, 2010; Hall and Mah, 2017). Biofilm models demonstrate that enzyme-responsive systems maintain antibiotic concentrations above the minimal biofilm eradication concentration (MBEC) for ≥24–48 h, whereas free antibiotics fall below MBEC within hours. In intracellular infections, enzymatic activation within pathogen-containing compartments enhances intracellular drug availability without increasing host-cell toxicity. Cytotoxicity assays consistently show >85–90% host-cell viability at therapeutically effective doses, underscoring the safety advantage of pathogen-activated release (Hofer, 2021). Enzyme-responsive nanocarriers redefine antimicrobial targeting by converting pathogen-derived enzymes into biochemical release (Chen et al., 2026). Their ability to couple drug activation directly to bacterial presence enables high specificity, minimizes off-target exposure, and provides a rational strategy to suppress resistance development. Within the broader classification of stimuli-responsive systems, enzyme-responsive platforms represent the most biologically discriminating approach to precision antimicrobial delivery (Sobczak, 2022).

### 3.3 ROS-responsive nanocarriers

Reactive oxygen species responsive nanocarriers represent a mechanistically distinct class of stimuli-responsive systems that exploit infection- and inflammation-associated oxidative stress as a trigger for antimicrobial release. Unlike pH- or enzyme-responsive platforms, which rely on localized physicochemical or pathogen-specific biochemical cues, ROS-responsive systems are activated primarily by host-driven immune responses, making them particularly relevant for severe, disseminated, and chronic infections characterized by intense inflammation (Hofer, 2021).

#### 3.3.1 Mechanistic principles

The defining feature of ROS-responsive nanocarriers is the incorporation of oxidation-sensitive chemical motifs that undergo rapid structural degradation in ROS-rich environments while remaining stable under physiological redox conditions. Two linker chemistries dominate current designs: thioketal and boronic ester linkers. Thioketal linkers are cleaved selectively by ROS such as hydrogen peroxide, hydroxyl radicals, and peroxynitrite. Under physiological conditions (ROS concentrations typically <10^−9^ M), thioketal-containing nanocarriers exhibit minimal degradation, maintaining >85–90% structural integrity over 48–72 h. In contrast, under inflammatory ROS levels (10^−7^–10^−6^ M), linker cleavage accelerates dramatically, with reported half-lives of 1–4 h, resulting in 65–90% drug release within 24 h. This sharp redox-dependent contrast enables robust on–off control of antimicrobial release. Boronic ester linkers provide an alternative ROS-sensitive mechanism, undergoing oxidation to phenols in the presence of hydrogen peroxide (Graham-Gurysh et al., 2020). These linkers demonstrate slightly faster cleavage kinetics than thioketals, with 50–70% payload release occurring within 6–12 h at ROS concentrations representative of acute inflammation, while remaining largely intact (>80% stability) in non-inflamed tissues. Importantly, both linker types can be integrated into polymer backbones, micelles, or crosslinked nanogels, allowing broad material flexibility (Rinaldi et al., 2022). A critical feature of ROS-responsive systems is their inflammation-driven release behavior. Because ROS production scales with immune activation, drug release rates correlate with the intensity of the inflammatory response (Pandey and Pandey, 2025). Quantitative in-vivo imaging studies show that ROS-responsive nanocarriers achieve 2–5-fold higher drug accumulation in inflamed infection sites compared with passive nanocarriers, despite comparable systemic exposure (Mi, 2020). In ROS-responsive systems, thioketal-based carriers maintain >85–90% structural integrity for 48–72 h under basal ROS conditions, but undergo rapid cleavage under inflammatory ROS concentrations (10^−7^–10^−6^ M), producing 65–90% drug release within 24 h. Boronic ester platforms often display faster kinetics, with 50–70% release within 6–12 h under hydrogen peroxide-rich conditions (Rinaldi et al., 2022). These findings confirm that oxidative stress can function as a quantitative release trigger rather than a qualitative concept alone.

#### 3.3.2 Therapeutic relevance

ROS-responsive nanocarriers are particularly relevant in sepsis, where systemic and organ-specific oxidative stress is a defining pathological feature. In experimental sepsis models, ROS levels in affected organs increase by one to two orders of magnitude, creating a permissive environment for redox-triggered drug release. ROS-responsive antibiotic delivery has demonstrated enhanced antibacterial efficacy in preclinical models, often accompanied by reduced inflammatory tissue injury and improved healing outcomes compared with free antibiotics or non-responsive controls. By coupling infection-triggered release with modulation of oxidative stress, these systems may improve therapeutic selectivity in inflamed tissues (Chen et al., 2021). In chronic inflammatory infections, such as osteomyelitis and implant-associated infections, persistent immune activation maintains elevated ROS levels even when bacterial burden fluctuates. ROS-responsive systems exploit this sustained oxidative milieu to provide prolonged, localized antimicrobial exposure. Release studies in chronic infection models demonstrate maintenance of drug concentrations above therapeutic thresholds for ≥48 h, whereas non-responsive systems exhibit rapid drug depletion within 12–24 h (Seebach and Kubatzky, 2019). Pulmonary infections, including bacterial pneumonia and cystic fibrosis–associated infections, represent another high-impact application. Inflamed lung tissue exhibits elevated ROS production from recruited neutrophils and macrophages, particularly in the alveolar space (Malainou et al., 2023). Aerosolized ROS-responsive nanocarriers have shown 3–4-fold higher pulmonary drug retention and significantly reduced systemic exposure compared with free antibiotics, translating into improved bacterial clearance with reduced nephrotoxicity and ototoxicity risk (Hadianamrei et al., 2022).

#### 3.3.3 Advantages and limitations

A key advantage of ROS-responsive nanocarriers is their ability to integrate antimicrobial therapy with inflammatory disease activity, enabling drug release that adapts dynamically to infection severity. This property is particularly advantageous in heterogeneous or disseminated infections where local pH or enzymatic activity may vary (Ballance et al., 2019; Lu et al., 2023a). However, ROS responsiveness is inherently host-dependent, and excessive oxidative stress may accelerate carrier degradation beyond optimal release kinetics, potentially leading to burst release. Moreover, sterile inflammatory conditions can also generate elevated ROS, raising the possibility of off-target activation. These limitations have motivated the development of dual-responsive systems that combine ROS sensitivity with pathogen-specific triggers, discussed in subsequent sections. ROS-responsive nanocarriers leverage infection-associated oxidative stress as a quantitative release cue, enabling inflammation-synchronized antimicrobial delivery. Their particular suitability for sepsis, chronic inflammatory infections, and lung infections positions them as a critical component of the stimuli-responsive nanocarrier landscape. ROS-responsive systems uniquely bridge antimicrobial delivery and immunopathology, offering therapeutic advantages that extend beyond pathogen eradication alone.

### 3.4 Multi-stimuli-responsive nanocarriers

Single-stimulus-responsive nanocarriers have demonstrated clear advantages in infection-targeted drug delivery; however, their performance is often constrained by the spatial and temporal heterogeneity of infection microenvironments. In clinically relevant settings, pathological cues such as acidosis, elevated reactive oxygen species (ROS), and bacterial enzyme activity coexist and fluctuate dynamically within biofilms, abscesses, and intracellular compartments. This heterogeneity can lead to incomplete or asynchronous activation of single-trigger systems, thereby limiting therapeutic precision (Flemming and Wingender, 2010). Multi-stimuli-responsive nanocarriers are designed to integrate two or more orthogonal triggers, enabling conditional or sequential drug release that better reflects the complexity of infection niches. Common configurations include pH/enzyme dual-responsive systems, in which acid-induced carrier destabilization enhances accessibility of enzyme-cleavable linkers, and pH/ROS-responsive platforms that couple acidosis-driven structural transitions with oxidation-sensitive bond cleavage (Mura et al., 2013; Ahmad et al., 2026). These combinatorial designs improve release selectivity by requiring the simultaneous presence of multiple pathological cues, thereby reducing premature drug leakage in non-infected tissues. Mechanistically, multi-stimuli systems frequently employ hierarchical or logic-gated activation strategies. In sequential (cascade) designs, one stimulus such as acidic pH induces conformational changes or charge reversal that enhance penetration into biofilms or cellular compartments, while a second trigger such as enzymatic cleavage or ROS-mediated bond disruption initiates final drug release. This two-stage activation has been shown to enhance intrabiofilm penetration and intracellular delivery compared with single-stimulus systems, particularly in environments characterized by diffusion barriers and metabolic gradients (Liang and Liu, 2016; Deiss-Yehiely et al., 2023). From a translational perspective, multi-stimuli-responsive nanocarriers offer a strategy to address inter-patient and intra-lesional variability in trigger intensity. By integrating multiple activation pathways, these systems reduce dependence on any single microenvironmental parameter, thereby improving robustness across diverse infection phenotypes, including chronic biofilm infections and intracellular persistence. However, increased structural complexity introduces challenges related to reproducibility, trigger threshold tuning, and large-scale manufacturing, which remain key barriers to clinical translation (Anselmo and Mitragotri, 2019). The multi-stimuli-responsive nanocarriers represent an evolution from single-trigger designs toward logic-controlled therapeutic systems that more accurately align drug activation with the multifactorial nature of infectious disease microenvironments.

### 3.5 Comparison of passive and stimuli-responsive nanocarriers

Passive nanocarriers primarily rely on nonspecific accumulation mechanisms, including enhanced permeability and retention (EPR)-like effects in inflamed tissues, to improve systemic pharmacokinetics relative to free antibiotics. While such systems can prolong circulation time and increase tissue exposure, drug release typically remains diffusion-driven and decoupled from local infection biology, resulting in limited control over spatial and temporal drug availability (Fang et al., 2011; Blanco et al., 2015). By contrast, stimuli-responsive nanocarriers are engineered to couple drug release to infection-associated cues including acidosis, bacterial enzymatic activity, and oxidative stress thereby enabling conditional activation within pathological microenvironments. This trigger-dependent behavior allows antimicrobial payloads to remain largely sequestered during systemic circulation while undergoing rapid release at sites of infection (Mura et al., 2013; Gao and Xiong, 2021). The pharmacodynamic implications of this distinction are substantial. Passive delivery may still result in sub-inhibitory antibiotic exposure in non-infected tissues and commensal microbiota, a condition shown to promote resistance selection, mutagenesis, and horizontal gene transfer even at concentrations well below the minimal inhibitory concentration (Gullberg et al., 2011). In contrast, stimuli-responsive systems are designed to maintain localized drug concentrations above bactericidal thresholds within infection sites, while minimizing systemic exposure and collateral selection pressure. These advantages are particularly relevant in pathophysiological contexts characterized by diffusion barriers and microenvironmental gradients, such as biofilms, intracellular infections, and poorly vascularized tissues, where passive accumulation alone is insufficient to achieve therapeutic concentrations (Thakur et al., 2019). However, the performance of stimuli-responsive systems is inherently dependent on the presence, magnitude, and spatial distribution of triggering cues, which may vary substantially across patients, anatomical sites, and stages of infection.

While passive nanocarriers improve systemic drug distribution, stimuli-responsive systems offer a higher level of pharmacological precision by aligning drug release with disease-specific conditions. Whether this added complexity translates into consistent clinical benefit remains contingent on overcoming variability in trigger intensity, manufacturing reproducibility, and regulatory validation.

## 4 Nanocarrier platforms used

**Figure 4:**
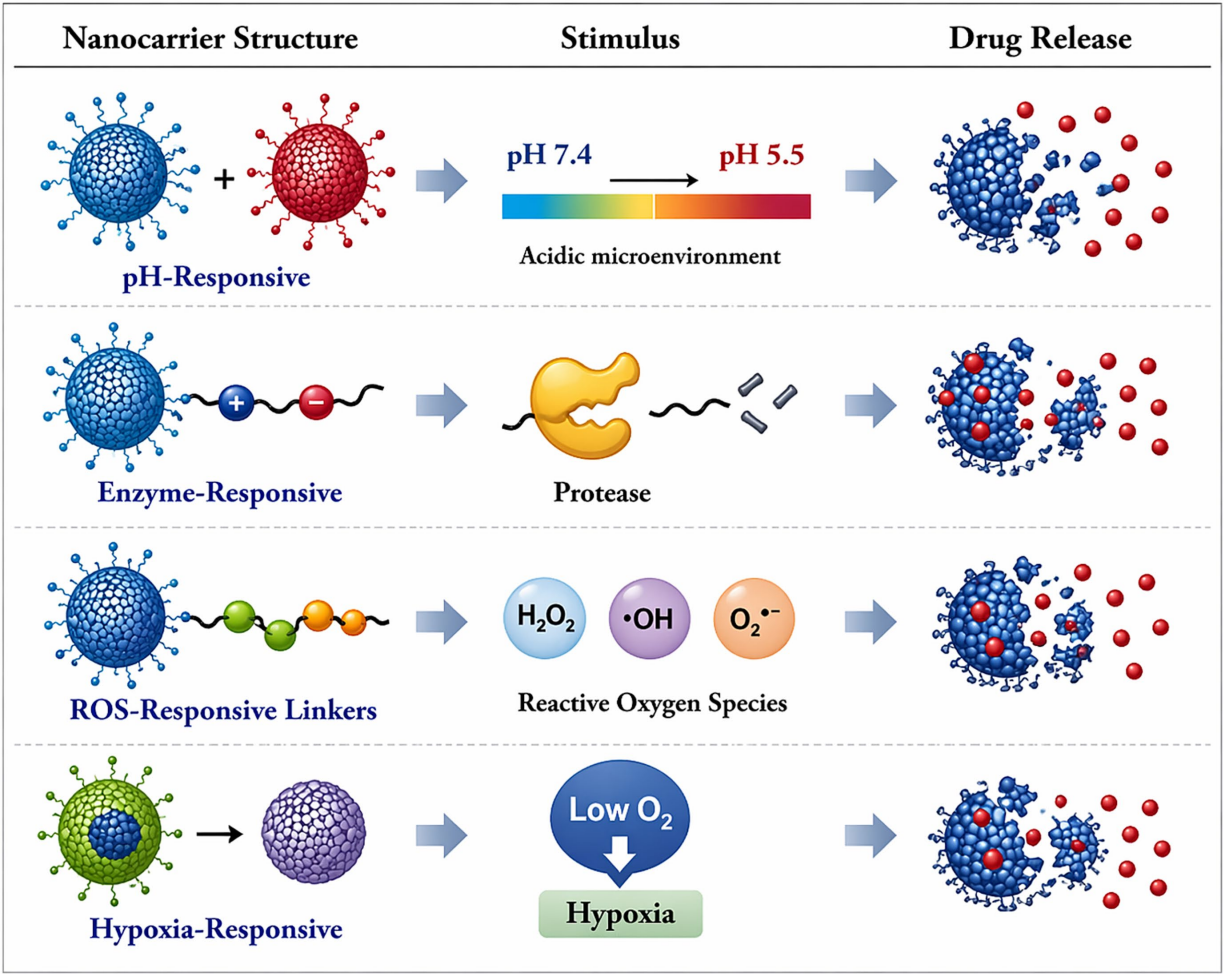
Stimuli-responsive nanocarrier platforms for infection-targeted antimicrobial delivery. pH-responsive nanocarriers undergo structural or charge changes under acidic conditions (pH ~5.5) typical of infected tissues, leading to drug release. Enzyme-responsive nanocarriers exploit pathogen-derived enzymes, such as proteases, to cleave functional linkers and trigger antimicrobial release. ROS-sensitive nanocarriers respond to elevated levels of reactive oxygen species (e.g., H_2_O_2_, OH, O_2_^−^) at inflamed or infected sites, resulting in oxidative linker degradation and drug liberation. Hypoxia-responsive nanocarriers are activated under low-oxygen conditions characteristic of chronic and biofilm-associated infections, enabling targeted antimicrobial delivery.

The effectiveness of stimuli-responsive antimicrobial delivery is critically determined by the physicochemical properties of the nanocarrier platform, which dictate drug loading efficiency, stimulus sensitivity, release kinetics, biodistribution, and safety (Blanco et al., 2015). While multiple material classes have been explored, only a subset combines stimulus integration, antimicrobial compatibility, and translational feasibility (Table 4). Their major stimulus-triggered release logic is schematically illustrated in Figure 4.

**Table 4:** Major nanocarrier platforms used for stimuli-responsive antimicrobial delivery.

Nanocarrier platform	Key material characteristics	Compatible stimuli	Advantages for antimicrobial delivery	Translational considerations
Polymeric nanoparticles/nanogels (Kamaly et al., 2016)	Synthetic or natural polymers [PLGA, PEG, chitosan, poly (β-amino esters)]	pH, enzyme, ROS, hypoxia	High drug loading, tunable degradation, versatile chemistry for stimulus integration	Scalable manufacturing; several FDA-approved polymers
Polymeric micelles (Cabral and Kataoka, 2014)	Amphiphilic block copolymers forming core–shell structures	pH, ROS, hypoxia	Efficient encapsulation of hydrophobic antibiotics; rapid stimulus-triggered disassembly	Stability in circulation must be optimized
Liposomes (stimuli-responsive) (Naseri et al., 2015)	Phospholipid bilayers with functionalized lipids	pH, enzyme, ROS	Excellent biocompatibility; established clinical history	Limited stability without surface modification
Solid lipid nanoparticles (SLNs) and nanostructured lipid carriers (NLCs) (Jacob et al., 2025)	Solid or mixed solid–liquid lipid matrices	pH, enzyme, ROS	Good physical stability; suitable for inhalation and topical delivery	Limited loading of hydrophilic drugs
Mesoporous silica nanoparticles (MSNs) (Tang et al., 2023)	Rigid porous silica frameworks with gated pores	pH, enzyme, ROS	High surface area; precise pore-gating control	Long-term biodegradability requires evaluation
Metal–organic frameworks (MOFs) (Abánades Lázaro et al., 2024)	Crystalline metal–ligand networks	pH, enzyme, redox	Extremely high loading capacity; programmable degradation	Early translational stage; metal toxicity considerations
Inorganic nanoparticles (gold, iron oxide) (Pelgrift and Friedman, 2013)	Metallic or magnetic cores with functional shells	ROS, enzyme, external stimuli	Imaging-guided delivery; magnetic targeting possible	Clearance and long-term accumulation must be addressed
Hybrid nanocarriers (Elhassan et al., 2022)	Polymer–lipid or organic–inorganic composites	Multi-stimuli (pH + enzyme + ROS)	Combines stability, responsiveness, and targeting	Increased formulation complexity

### 4.1 Polymeric nanoparticles

Polymeric nanoparticles most commonly based on poly (lactic-co-glycolic acid) (PLGA) and chitosan represent the most extensively studied antimicrobial delivery platforms. PLGA nanoparticles typically achieve drug encapsulation efficiencies of 50–80%, with degradation-controlled release spanning 24 h to >7 days, depending on polymer composition and molecular weight (Alsaab et al., 2022; Jumadi et al., 2025). Their hydrolytic degradation products are biocompatible, supporting clinical translation. Chitosan-based systems offer additional advantages due to their intrinsic pH responsiveness and cationic charge, which enhances bacterial membrane interaction. Quantitative uptake studies demonstrate 2–4-fold higher bacterial binding for chitosan nanoparticles compared with neutral polymeric carriers. However, batch-to-batch variability and limited stability under physiological conditions remain challenges (Desai et al., 2023; Karimlar et al., 2026). Polymeric platforms readily integrate pH-, enzyme-, and ROS-sensitive linkers within their backbone or crosslinkers, enabling modular stimulus-responsive design.

### 4.2 Liposomes and lipid nanoparticles

Liposomes and lipid nanoparticles (LNPs) are clinically validated carriers with well-established manufacturing pipelines. Conventional liposomes typically encapsulate 20–40% of hydrophilic antibiotics, whereas optimized formulations can exceed 60% loading for amphiphilic drugs (Mehta et al., 2023; Basak and Das, 2025). Stimuli responsiveness is introduced through pH-sensitive lipids, enzyme-cleavable coatings, or ROS-labile lipid components. Quantitative release studies indicate that pH-responsive liposomes can achieve >70% drug release within 12 h at pH ≤ 6.0, compared with <20% at pH 7.4, supporting infection-site selectivity (Lee and Thompson, 2017). Lipid platforms also exhibit favourable pharmacokinetics, reducing renal clearance and achieving 2–3-fold higher tissue retention in infected organs. However, premature leakage and limited stability in inflammatory environments may constrain their use in severe infections unless reinforced by secondary stabilizing layers (Jacob et al., 2025).

### 4.3 Mesoporous silica nanoparticles (MSNs)

Mesoporous silica nanoparticles offer exceptionally high surface area (>800 m^2^ g^−1^) and pore volumes that enable drug loading efficiencies exceeding 80–90% (Castillo et al., 2019). Their rigid framework supports precise pore gating using stimulus-responsive “caps,” including pH-cleavable polymers or enzyme-sensitive molecular valves. Release kinetics from MSNs can be tightly controlled, with studies reporting **<**10% premature release under physiological conditions and >80% release upon stimulus activation. MSNs are particularly effective for combination antimicrobial delivery, although long-term biodegradation and accumulation concerns persist. MSNs exemplify structure-encoded stimulus responsiveness, where release is governed by pore opening rather than carrier degradation (Castillo et al., 2019; Almatroudi, 2025).

### 4.4 Metal–organic frameworks (MOFs)

Metal–organic frameworks (MOFs) represent an emerging class of antimicrobial nanocarriers characterized by high porosity and tunable coordination chemistry. Many MOF platforms demonstrate substantial drug-loading capacity, while their metal–ligand bonds can be engineered to dissociate under acidic or redox conditions, enabling stimuli-responsive cargo release (Cai et al., 2022; Benny et al., 2024). Importantly, certain MOFs provide dual functionality, combining controlled drug release with intrinsic antimicrobial activity via metal ion release. Quantitative infection models demonstrate >60% bacterial burden reduction using MOF-based systems at doses where free antibiotics show minimal efficacy (Zheng et al., 2025). Nevertheless, concerns regarding metal toxicity, stability in biological fluids, and scalable synthesis currently limit clinical translation.

### 4.5 Biomimetic Nanocarriers

Biomimetic systems particularly cell membrane-coated nanoparticles represent the most biologically sophisticated platform. By cloaking nanoparticles with erythrocyte, platelet, or bacterial membranes, these systems achieve immune evasion and prolonged circulation, with circulation half-lives increased by 2–5-fold compared with uncoated particles (Xia et al., 2023). Biomimetic carriers also exhibit enhanced infection targeting, with reported 3–6-fold increases in accumulation at infected tissues. When combined with stimulus-responsive cores, they integrate biological camouflage with controlled release (Alvarez-Lorenzo et al., 2025). However, complexity of fabrication and regulatory uncertainty remain significant barriers.

## 5 Role of stimuli-responsive nanocarriers in overcoming antimicrobial resistance

Beyond improving pharmacokinetics, stimuli-responsive nanocarriers directly address mechanistic drivers of antimicrobial resistance by reshaping drug–pathogen–host interactions. Their impact extends beyond enhanced delivery to evolutionary pressure modulation.

### 5.1 Bypassing efflux pumps

Efflux pumps reduce intracellular antibiotic concentrations by 10–100-fold, particularly in Gram-negative bacteria (Zhang et al., 2024). Nanocarrier-mediated delivery circumvents this mechanism by promoting endocytic or membrane-fusion-based uptake, enabling antibiotics to enter bacterial cells independently of transporter systems (Rajkhowa et al., 2024). Quantitative studies demonstrate 2–6-fold increases in intracellular antibiotic accumulation, restoring susceptibility in efflux-dominant resistant strains (Li et al., 2015).

### 5.2 Protection from enzymatic degradation

Many resistance mechanisms rely on enzymatic drug inactivation, such as β-lactamase-mediated hydrolysis (Mora-Ochomogo and Lohans, 2021). Encapsulation within nanocarriers can physically shield antibiotics from premature enzymatic degradation, thereby prolonging functional activity in enzyme-rich infection environments. When combined with enzyme-triggered release, this strategy may convert resistance-associated enzymes from therapeutic barriers into selective activators of localized drug release (Torres-Herrero et al., 2024).

### 5.3 Sustained supra-MIC exposure

Resistance evolution is strongly driven by sub-inhibitory antibiotic exposure, which promotes mutagenesis, biofilm formation, and horizontal gene transfer (Sharif and Yadav, 2025). Stimuli-responsive systems maintain localized drug concentrations above the MIC or MBEC for ≥24–48 h, compared with rapid decline below MIC within hours for free antibiotics (Hindieh et al., 2025). This sustained exposure significantly enhances bacterial killing and reduces adaptive responses. Beyond pharmacokinetic advantages, several studies report direct improvements in apparent antimicrobial potency. Representative examples include pH-responsive imipenem nanocarriers against *Acinetobacter baumannii*, where MIC values decreased by approximately 4–8-fold compared with free imipenem. Enzyme-triggered systems delivering β-lactams or aminoglycosides have shown 2–6-fold reductions in MIC against resistant Enterobacterales and *Pseudomonas aeruginosa*. In mature biofilm models, the minimum biofilm eradication concentration (MBEC) or bactericidal thresholds are frequently reduced by 1–2log^₁₀^ equivalent doses, reflecting enhanced local delivery and sustained supra-MIC exposure (Zhou et al., 2022). Where measured, MBC values also decline substantially, particularly in intracellular infection models where free-drug access is otherwise limited.

### 5.4 Reduction of horizontal gene transfer pressure

Sub-MIC antibiotic levels increase conjugation frequencies by up to 10-fold in mixed bacterial populations. By confining antimicrobial exposure to infected sites and avoiding systemic low-dose dissemination, controlled-release nanocarriers reduce selective pressure on commensal microbiota, thereby suppressing resistance gene propagation (Flores-Vargas et al., 2023). Stimulus-triggered release fundamentally alters the evolutionary landscape of antimicrobial therapy. By avoiding sub-inhibitory exposure and aligning drug activation with infection-specific cues, stimuli-responsive nanocarriers suppress the very conditions under which resistance emerges, offering benefits that extend beyond immediate antibacterial efficacy (Bag et al., 2023).

## 6 Targeted applications in drug-resistant infections

Stimuli-responsive nanocarriers demonstrate their particular therapeutic value in infection contexts where conventional antibiotics systematically fail due to pharmacokinetic, biological, and resistance-associated barriers. Preclinical evidence increasingly supports their application across diverse drug-resistant infection phenotypes. Representative case studies further illustrate the therapeutic gains achievable with infection-responsive delivery. Nanocarrier-based delivery systems have been reported to enhance antibiotic efficacy in multidrug-resistant Gram-negative infections alongside reduced systemic toxicity (Karaiskos and Giamarellou, 2014). In chronic lung infections caused by *Pseudomonas aeruginosa*, nanoparticle-based delivery systems have demonstrated improved penetration into biofilms and enhanced antimicrobial activity compared with free antibiotics, particularly in cystic fibrosis–associated infections (Filkins and O’Toole, 2015). For intracellular infections, nanocarriers capable of targeting phagocytic cells have been shown to increase intracellular antibiotic accumulation and improve bacterial clearance in pathogens such as *Salmonella enterica* and *Mycobacterium tuberculosis* (Thakur et al., 2019). surface-engineered and responsive nanocarrier systems have demonstrated localized antimicrobial activity at biomaterial interfaces, reducing bacterial colonization while maintaining tissue compatibility (Ding et al., 2022). Across these examples, the recurring advantage is not merely nanoparticle encapsulation itself, but the ability to enhance local drug exposure and improve therapeutic efficacy in challenging infection microenvironments.

### 6.1 MDR gram-negative bacterial infections

Multidrug-resistant (MDR) Gram-negative pathogens, including *Acinetobacter baumannii*, *Pseudomonas aeruginosa*, and *Klebsiella pneumoniae*, present formidable therapeutic challenges due to low membrane permeability, efflux pump overexpression, and enzymatic drug degradation. In murine thigh and lung infection models, pH- and stimulus-responsive colistin or carbapenem nanocarriers in murine thigh and lung models have produced substantially lower bacterial burdens in preclinical models at doses that were subtherapeutic in free-drug form, while reducing systemic toxicity markers. Importantly, these systems reduced nephrotoxicity markers by 30–40%, underscoring the benefit of localized delivery (Karaiskos and Giamarellou, 2014; Cillóniz et al., 2019; Mukhopadhyay et al., 2025).

### 6.2 Biofilm-associated infections

Biofilms are responsible for an estimated 65–80% of chronic bacterial infections, including catheter-, prosthesis-, and wound-associated infections. Animal biofilm models consistently show that stimuli-responsive nanocarriers penetrate biofilm matrices more effectively than free drugs or passive nanoparticles (Haval et al., 2025). Charge-reversal pH-responsive systems demonstrate 30–50% greater biofilm penetration depth, while enzyme-responsive platforms maintain antibiotic concentrations above the minimal biofilm eradication concentration (MBEC) for ≥24–48 h. In mature biofilm models, charge-reversal systems have demonstrated improved antibacterial activity compared with conventional treatment, largely due to enhanced biofilm penetration and sustained local drug exposure above minimum biofilm eradication concentration (MBEC) thresholds. The magnitude of benefit varies across formulations, pathogens, and experimental models (Sun et al., 2025).

### 6.3 Intracellular pathogens

Intracellular pathogens such as *Mycobacterium tuberculosis*, *Salmonella enterica*, and *Listeria monocytogenes* evade antibiotic exposure by residing within host cells (Thakur et al., 2019). In macrophage infection models, stimuli-responsive nanocarriers have shown higher intracellular antibiotic accumulation, improved bacterial killing, and preserved host-cell viability compared with free antibiotics. These findings support their potential for infections where cellular penetration is a major therapeutic barrier (Hosseini et al., 2022). Cellular uptake studies further demonstrate that stimuli-responsive systems significantly improve intracellular delivery.

### 6.4 Pulmonary and wound infections

Pulmonary infections, particularly in cystic fibrosis and ventilator-associated pneumonia, are characterized by intense inflammation and oxidative stress. Aerosolized ROS-responsive nanocarriers demonstrate 3–4-fold prolonged lung retention and improved bacterial clearance relative to inhaled free antibiotics (Filkins and O’Toole, 2015). Similarly, in chronic wound infection models, stimuli-responsive delivery enhances local drug exposure while reducing systemic absorption, accelerating wound closure by 25–40% compared with standard care (Kumar et al., 2023).

### 6.5 Sepsis and implant-associated infections

In sepsis, where systemic antibiotic exposure often exacerbates organ toxicity, inflammation-responsive nanocarriers reduce bacterial burden by 40–60% while attenuating cytokine-mediated tissue damage (Lu et al., 2023b). For implant-associated infections, biomimetic and enzyme-responsive systems localize drug release at the biomaterial interface, reducing bacterial colonization by >70% in rodent models without impairing implant integration (Ding et al., 2020).

## 7 Safety, pharmacokinetics, and biodistribution

Despite their therapeutic promise, the clinical translation of stimuli-responsive nanocarriers hinges on rigorous evaluation of safety and *in-vivo* behavior. Biodegradable polymeric carriers typically degrade over days to weeks, while lipid-based systems clear within hours to days, depending on composition (Das et al., 2020). Controlled degradation is essential to avoid premature drug release or long-term tissue accumulation. Quantitative biodistribution studies show that optimized nanocarriers achieve 2–3-fold higher accumulation at infection sites while maintaining low residual levels in liver and spleen after 72 h. Surface chemistry strongly influences immune recognition. Biomimetic coatings and PEGylation reduce opsonization, extending circulation half-life by 2–5-fold (Perrigue et al., 2021). However, repeated administration can trigger immune memory or complement activation, necessitating careful immunotoxicity assessment in chronic infection models. Nanocarrier size dictates clearance routes: particles <10 nm undergo rapid renal elimination, whereas larger systems are cleared via the mononuclear phagocyte system. Stimuli-responsive designs must balance prolonged circulation with efficient post-therapy clearance to avoid organ accumulation. Long-term toxicity data remain limited. In subchronic rodent studies (4–12 weeks), most polymeric and lipid nanocarriers show no significant histopathological abnormalities, whereas certain inorganic systems exhibit dose-dependent accumulation. These findings underscore the importance of material choice for chronic or prophylactic applications. Beyond acute tolerability, long-term safety depends strongly on carrier composition, degradation kinetics, cumulative dose, and frequency of administration. Biodegradable systems such as PLGA, chitosan, and many lipid nanoparticles are generally metabolized into endogenous or excretable products, reducing the probability of persistent tissue retention. Nevertheless, incomplete degradation or repeated dosing may still impose chronic burden on the reticuloendothelial system, particularly in the liver and spleen. By contrast, slowly degradable inorganic platforms including some silica-, metallic-, or hybrid nanomaterials may persist for prolonged periods after repeated exposure, raising concerns regarding oxidative stress, fibrosis, altered macrophage activity, or delayed organ toxicity not captured in short-term studies. These considerations indicate that chronic-use applications require extended biodistribution, recovery, and repeat-dose toxicology studies rather than reliance on acute endpoints alone (Blanco et al., 2015; Germain et al., 2020). Although many formulations are engineered for immune compatibility, unwanted immunostimulation remains a clinically relevant concern. Particle size, shape, surface charge, residual solvents, endotoxin contamination, and adsorbed plasma proteins can all influence complement activation, cytokine release, and leukocyte recruitment. Cationic carriers may enhance membrane perturbation and inflammatory signaling, whereas repeated administration of PEGylated systems can in some cases provoke accelerated blood clearance or anti-PEG immune responses. In susceptible patients, these events could manifest as infusion reactions, hypersensitivity, exaggerated local inflammation, or altered pharmacokinetics during subsequent dosing cycles. Therefore, comprehensive safety assessment should include complement activation testing, cytokine profiling, hemocompatibility, pyrogenicity, anti-drug/anti-carrier antibody monitoring, and repeated-dose immunotoxicology (Moghimi et al., 2011). From a translational perspective, favorable pre-clinical safety does not always guarantee clinical success. Several nanomedicine programs have demonstrated promising antimicrobial or targeting efficacy experimentally but encountered challenges during human development because of manufacturing variability, unstable release behavior, modest therapeutic benefit over standard therapy, or emergent safety signals. For infection-responsive systems, additional uncertainties include heterogeneous trigger intensity across patients, unpredictable release in sepsis or organ dysfunction, and difficulty identifying trial endpoints that capture both microbiological clearance and host recovery. Future clinical studies should therefore integrate pharmacokinetic/pharmacodynamic modeling, biomarker-guided patient selection, long-term follow-up, and direct comparison with optimized standard-of-care regimens to establish a robust benefit–risk profile (Hare et al., 2017; Anselmo and Mitragotri, 2019).

## 8 Translational challenges and regulatory considerations

The path from laboratory innovation to clinical implementation remains nontrivial. Scale-up reproducibility is hindered by complex synthesis protocols, particularly for multi-stimuli and biomimetic systems. Batch-to-batch variability can alter release kinetics by >20–30%, raising concerns for regulatory approval. Regulatory classification presents another challenge: stimuli-responsive nanocarriers do not fit neatly into existing drug or device categories, complicating safety evaluation and approval pathways. Moreover, the lack of standardized infection models limits cross-study comparability and hampers evidence consolidation (Anselmo and Mitragotri, 2019). A further obstacle is the absence of harmonized protocols for evaluating stimuli-responsive performance across laboratories. Published studies frequently employ different trigger intensities, buffer systems, serum conditions, biofilm maturity stages, bacterial inocula, and release assay methods, making direct comparison difficult. For instance, “acidic conditions” may vary from pH 6.8 to pH 5.0, while ROS-triggered systems are tested using widely different peroxide concentrations or cellular inflammatory models. Likewise, efficacy endpoints may be expressed as MIC, MBC, MBEC, log₁₀ CFU reduction, biomass loss, fluorescence uptake, or survival benefit without consistent benchmarking against free-drug and non-responsive controls. Consensus standards for physicochemical characterization, trigger-threshold definition, release kinetics under biologically relevant conditions, serum stability, batch reproducibility, and core microbiological endpoints would substantially improve reproducibility and regulatory confidence (Faria et al., 2018; Germain et al., 2020).

Clinical precedents support the feasibility of nanomedicine translation, even though infection-responsive systems remain at an earlier stage than oncology nanotherapeutics. ThermoDox® (heat-triggered liposomal doxorubicin), evaluated in phase-III studies, demonstrated that externally triggered release systems can be manufactured at scale and tested under regulatory oversight (Dou et al., 2017; Lyon et al., 2018). Likewise, pH-sensitive liposomal formulations and enzyme-degradable polymeric carriers have advanced into human studies in oncology and inflammatory diseases, establishing regulatory pathways for stimulus-activated materials (Cabral and Kataoka, 2014). In infectious diseases, approved liposomal antimicrobials such as liposomal amphotericin B (AmBisome®) and inhaled liposomal amikacin (Arikayce®) do not employ sophisticated endogenous triggers, but they validate the clinical utility of nanocarrier-mediated targeting, toxicity reduction, and controlled tissue exposure (Stone et al., 2016; Olivier et al., 2017). These products provide an important translational foundation upon which next-generation infection-responsive nanocarriers can build. Despite strong preclinical efficacy, the clinical conversion rate of advanced nanocarriers remains low. Several factors explain this gap. First, infection microenvironments are highly heterogeneous across patients, pathogens, and anatomical sites; a trigger such as acidity or ROS may be robust in one model but weak or transient in another. Second, many systems rely on complex multicomponent architectures that are difficult to reproduce during scale-up, resulting in batch variability in size, loading efficiency, and release kinetics. Third, animal infection models frequently fail to replicate human pharmacokinetics, immune responses, chronic biofilm biology, or comorbid states such as diabetes and renal dysfunction. Fourth, regulatory expectations for combination products require parallel demonstration of material safety, drug performance, degradation profiles, and manufacturing consistency. Finally, commercial barriers including high production cost, uncertain reimbursement, and limited superiority over generic antibiotics reduce industrial investment. Collectively, these scientific, regulatory, and economic barriers explain why many elegant laboratory systems do not progress to clinical trials (Hare et al., 2017; Germain et al., 2020). Registration of “smart” nanocarriers introduces additional challenges because approval depends not only on composition but also on dynamic function. Regulators may require evidence that the carrier remains stable during storage and circulation, activates only under intended pathological conditions, and maintains predictable release behavior despite patient-to-patient variability. This expands the evidentiary burden to include trigger sensitivity, activation thresholds, degradation products, shelf-life under realistic storage conditions, and lot-to-lot consistency of responsiveness. Classification may also vary between drug, device, or combination-product pathways, particularly for externally activated systems or platforms co-delivering antimicrobial and immunomodulatory payloads. Early regulatory engagement and quality-by-design development strategies are therefore especially important for these next-generation systems (Bobo et al., 2016). Compared with currently available anti-infective nanomedicines, infection-responsive systems aim to move beyond passive drug encapsulation toward disease-synchronized release. AmBisome® primarily reduces amphotericin B toxicity and improves tolerability, while Arikayce® prolongs pulmonary residence time of amikacin through inhaled liposomal deposition. In contrast, pH-, enzyme-, or ROS-responsive antimicrobial nanocarriers are designed to release payloads preferentially within acidic biofilms, enzyme-rich bacterial niches, or inflamed tissues. This may provide higher local drug concentrations, lower systemic exposure, and reduced resistance-selective pressure. However, marketed products currently retain practical advantages including validated large-scale manufacturing, longer shelf-life, simpler quality control, and established clinical benefit. Therefore, future infection-responsive nanomedicines must demonstrate not only mechanistic sophistication but also clear superiority over existing approved formulations in efficacy, safety, cost-effectiveness, and real-world usability (Bulbake et al., 2017; Ventola, 2017). Scaling laboratory synthesis into commercial manufacturing remains particularly challenging because small process deviations can alter both particle attributes and trigger responsiveness. Variations in mixing rate, solvent removal, temperature, raw-material purity, polymer molecular weight, linker density, or surface functionalization may shift particle size distribution, encapsulation efficiency, zeta potential, and activation kinetics. A formulation optimized to release 80% payload at pH 5.5 in bench-scale production may lose selectivity after scale-up if nanoscale architecture changes during manufacturing. Biomimetic systems add further complexity related to membrane sourcing, biological variability, sterilization, and storage stability. To address these barriers, continuous manufacturing, in-line analytical monitoring, predefined critical quality attributes, and release assays that directly confirm retained responsiveness should be incorporated early in development (Germain et al., 2020). The future success of infection-responsive nanomedicine will depend not only on smarter trigger design, but on the ability to outperform clinically available formulations under realistic manufacturing and healthcare constraints.

## 9 Future directions

Future advances will likely emerge at the intersection of materials science, data science, and clinical microbiology. Artificial intelligence (AI)-guided stimulus selection may optimize trigger combinations based on infection phenotype, while personalized nanomedicine approaches could tailor carrier composition and responsiveness to patient-specific inflammatory, metabolic, or microbial profiles. Addressing microenvironmental heterogeneity will likely require such precision strategies rather than a one-size-fits-all formulation. Integration with rapid diagnostics may further enable theranostic platforms capable of sensing infection severity, pathogen burden, or local biochemical cues and adjusting drug release accordingly. Beyond carrier engineering alone, next-generation anti-infective strategies are increasingly converging with biological and genetic therapeutics. For example, CRISPR-based antimicrobials may enable sequence-specific elimination of resistance genes or pathogenic strains, while engineered bacteriophage therapy offers programmable bacterial targeting and self-amplifying antimicrobial activity. In parallel, immunomodulatory nanotherapies that regulate excessive inflammation or enhance host defense could improve outcomes in sepsis and chronic infection, and microbiome-directed therapies may help restore colonization resistance and reduce recurrent infection after antibiotic exposure. Stimuli-responsive nanocarriers could serve as enabling platforms for the targeted delivery of these advanced payloads. Finally, combining antimicrobial delivery with host-directed or precision biologic therapies offers a route to simultaneously eradicate pathogens, limit collateral damage, and restore immune homeostasis. Together, these developments position stimuli-responsive nanocarriers not merely as improved drug delivery vehicles, but as adaptive therapeutic systems capable of reshaping antimicrobial treatment paradigms in the era of escalating resistance.

## References

[ref1] Abánades Lázaro I. Chen X. Ding M. Eskandari A. Fairen-Jimenez D. Giménez-Marqués M. . (2024). Metal–organic frameworks for biological applications. Nat. Rev. Methods Primers 4:42. doi: 10.1038/s43586-024-00320-8

[ref2] Abdesselem M. Pétri N. Kuhner R. Mousseau F. Rouffiac V. Gacoin T. . (2023). Real-time in vivo ROS monitoring with luminescent nanoparticles reveals skin inflammation dynamics. Biomed. Opt. Express 14, 5392–5404. doi: 10.1364/BOE.501914, 37854553 PMC10581786

[ref3] Ahmad Q. Mehdi S. Shaukat B. Siddique R. Asif M. T. Malik A. . (2026). Multi-stimuli responsive nanoparticles: next-generation platforms for smart drug delivery. OpenNano 29:100296. doi: 10.1016/j.onano.2026.100296

[ref4] Ahmad-Mansour N. Loubet P. Pouget C. Dunyach-Remy C. Sotto A. Lavigne J.-P. . (2021). *Staphylococcus aureus* toxins: an update on their pathogenic properties and potential treatments. Toxins (Basel) 13:677. doi: 10.3390/toxins13100677, 34678970 PMC8540901

[ref5] Almatroudi A. (2025). Advances in mesoporous silica and hybrid nanoparticles for drug delivery: synthesis, functionalization, and biomedical applications. Pharmaceutics 17:1602. doi: 10.3390/pharmaceutics17121602, 41471116 PMC12736835

[ref6] Alsaab H. O. Alharbi F. D. Alhibs A. S. Alanazi N. B. Alshehri B. Y. Saleh M. A. . (2022). PLGA-based nanomedicine: history of advancement and development in clinical applications of multiple diseases. Pharmaceutics 14:2728. doi: 10.3390/pharmaceutics14122728, 36559223 PMC9786338

[ref7] Alvarez-Lorenzo C. Ramirez-Romero A. Peixoto D. Vivero-Lopez M. Rodríguez-Moldes I. Concheiro A. (2025). Biomimetic cell membrane-coated scaffolds for enhanced tissue regeneration. Adv. Mater. 37:e2507084. doi: 10.1002/adma.202507084, 40665933 PMC12510297

[ref8] Anselmo A. C. Mitragotri S. (2019). Nanoparticles in the clinic: an update. Bioeng. Transl. Med. 4:e10143. doi: 10.1002/btm2.10143, 31572799 PMC6764803

[ref9] Bae Y. Nishiyama N. Kataoka K. (2007). In vivo antitumor activity of the folate-conjugated pH-sensitive polymeric micelle selectively releasing adriamycin in the intracellular acidic compartments. Bioconjug. Chem. 18, 1131–1139. doi: 10.1021/bc060401p, 17488066

[ref10] Bag N. Bardhan S. Roy S. Roy J. Mondal D. Guo B. . (2023). Nanoparticle-mediated stimulus-responsive antibacterial therapy. Biomater. Sci. 11, 1994–2019. doi: 10.1039/D2BM01941H, 36748318

[ref11] Ballance W. C. Qin E. C. Chung H. J. Gillette M. U. Kong H. (2019). Reactive oxygen species-responsive drug delivery Systems for the Treatment of neurodegenerative diseases. Biomaterials 217:119292. doi: 10.1016/j.biomaterials.2019.119292, 31279098 PMC7081518

[ref12] Basak S. Das T. K. (2025). Liposome-based drug delivery systems: from laboratory research to industrial production—instruments and challenges. ChemEngineering 9:56. doi: 10.3390/chemengineering9030056

[ref13] Benny A. Kalathiparambil Rajendra Pai S. D. Pinheiro D. Chundattu S. J. (2024). Metal organic frameworks in biomedicine: innovations in drug delivery. Results Chem. 7:101414. doi: 10.1016/j.rechem.2024.101414

[ref14] Bjarnsholt T. Whiteley M. Rumbaugh K. P. Stewart P. S. Jensen P. Ø. Frimodt-Møller N. (2022). The importance of understanding the infectious microenvironment. Lancet Infect. Dis. 22, e88–e92. doi: 10.1016/S1473-3099(21)00122-5, 34506737 PMC9190128

[ref15] Blanco E. Shen H. Ferrari M. (2015). Principles of nanoparticle design for overcoming biological barriers to drug delivery. Nat. Biotechnol. 33, 941–951. doi: 10.1038/nbt.3330, 26348965 PMC4978509

[ref16] Blaser M. J. (2016). Antibiotic use and its consequences for the normal microbiome. Science 352, 544–545. doi: 10.1126/science.aad9358, 27126037 PMC4939477

[ref17] Bobo D. Robinson K. J. Islam J. Thurecht K. J. Corrie S. R. (2016). Nanoparticle-based medicines: a review of FDA-approved materials and clinical trials to date. Pharm. Res. 33, 2373–2387. doi: 10.1007/s11095-016-1958-5, 27299311

[ref18] Bulbake U. Doppalapudi S. Kommineni N. Khan W. (2017). Liposomal formulations in clinical use: an updated review. Pharmaceutics 9:12. doi: 10.3390/pharmaceutics9020012, 28346375 PMC5489929

[ref19] Bush K. Bradford P. A. (2020). Epidemiology of β-lactamase-producing pathogens. Clin. Microbiol. Rev. 33, e00047–e00019. doi: 10.1128/CMR.00047-19, 32102899 PMC7048014

[ref20] Cabral H. Kataoka K. (2014). Progress of drug-loaded polymeric micelles into clinical studies. J. Control. Release 190, 465–476. doi: 10.1016/j.jconrel.2014.06.042, 24993430

[ref21] Cai X. Bao X. Wu Y. (2022). Metal–organic frameworks as intelligent drug nanocarriers for cancer therapy. Pharmaceutics 14:2641. doi: 10.3390/pharmaceutics14122641, 36559134 PMC9781098

[ref22] Castillo R. R. Lozano D. González B. Manzano M. Izquierdo-Barba I. Vallet-Regí M. (2019). Advances in mesoporous silica nanoparticles for targeted stimuli-responsive drug delivery: an update. Expert Opin. Drug Deliv. 16, 415–439. doi: 10.1080/17425247.2019.1598375, 30897978 PMC6667337

[ref23] Chen L. Huang Q. Zhao T. Sui L. Wang S. Xiao Z. . (2021). Nanotherapies for sepsis by regulating inflammatory signals and reactive oxygen and nitrogen species: new insight for treating COVID-19. Redox Biol. 45:102046. doi: 10.1016/j.redox.2021.102046, 34174559 PMC8205260

[ref24] Chen D. Li D. Su L. Wang D.-Y. Ren Y. Mei H. C. van der (2026). Stimuli-responsive, antimicrobial-loaded nanocarriers for oral biofilm control and microbiome restoration. Int. J. Oral Sci. 18:17. doi: 10.1038/s41368-025-00422-3 41667443 PMC12891596

[ref25] Cillóniz C. Dominedò C. Torres A. (2019). Multidrug resistant gram-negative Bacteria in community-acquired pneumonia. Crit. Care 23:79. doi: 10.1186/s13054-019-2371-3, 30850010 PMC6408800

[ref26] Das S. S. Bharadwaj P. Bilal M. Barani M. Rahdar A. Taboada P. . (2020). Stimuli-responsive polymeric nanocarriers for drug delivery, imaging, and theragnosis. Polymers 12:1397. doi: 10.3390/polym12061397, 32580366 PMC7362228

[ref27] David S. Mentasti M. Parkhill J. Chalker V. J. (2018). Low genomic diversity of *Legionella pneumophila* within clinical specimens. Clin. Microbiol. Infect. 24, 1020.e1–1020.e4. doi: 10.1016/j.cmi.2018.03.004, 29549055 PMC6123502

[ref28] de la Rica R. Aili D. Stevens M. M. (2012). Enzyme-responsive nanoparticles for drug release and diagnostics. Adv. Drug Deliv. Rev. 64, 967–978. doi: 10.1016/j.addr.2012.01.002, 22266127

[ref29] Deirram N. Zhang C. Kermaniyan S. S. Johnston A. P. R. Such G. K. (2019). Ph-responsive polymer nanoparticles for drug delivery. Macromol. Rapid Commun. 40:e1800917. doi: 10.1002/marc.201800917, 30835923

[ref30] Deiss-Yehiely E. Cárcamo-Oyarce G. Berger A. G. Ribbeck K. Hammond P. T. (2023). pH-responsive, charge-reversing layer-by-layer nanoparticle surfaces enhance biofilm penetration and eradication. ACS Biomater. Sci. Eng. 9, 4794–4804. doi: 10.1021/acsbiomaterials.3c00481, 37390118 PMC11117027

[ref31] Deng W. Marshall N. C. Rowland J. L. McCoy J. M. Worrall L. J. Santos A. S. . (2017). Assembly, structure, function and regulation of type III secretion systems. Nat. Rev. Microbiol. 15, 323–337. doi: 10.1038/nrmicro.2017.20 28392566

[ref32] Desai N. Rana D. Salave S. Gupta R. Patel P. Karunakaran B. . (2023). Chitosan: a potential biopolymer in drug delivery and biomedical applications. Pharmaceutics 15:1313. doi: 10.3390/pharmaceutics15041313, 37111795 PMC10144389

[ref33] Dinarello C. A. (2000). Proinflammatory cytokines. Chest 118, 503–508. doi: 10.1378/chest.118.2.503, 10936147

[ref34] Ding Y. Hao Y. Yuan Z. Tao B. Chen M. Lin C. . (2020). A dual-functional implant with an enzyme-responsive effect for bacterial infection therapy and tissue regeneration. Biomater. Sci. 8, 1840–1854. doi: 10.1039/c9bm01924c, 31967110

[ref35] Ding H. Tan P. Fu S. Tian X. Zhang H. Ma X. . (2022). Preparation and application of pH-responsive drug delivery systems. J. Control. Release 348, 206–238. doi: 10.1016/j.jconrel.2022.05.056, 35660634

[ref36] Dou Y. Hynynen K. Allen C. (2017). To heat or not to heat: challenges with clinical translation of thermosensitive liposomes. J. Control. Release 249, 63–73. doi: 10.1016/j.jconrel.2017.01.025, 28122204

[ref37] Du J.-Z. Sun T.-M. Song W.-J. Wu J. Wang J. (2010). A tumor-acidity-activated charge-conversional nanogel as an intelligent vehicle for promoted tumoral-cell uptake and drug delivery. Angew. Chem. Int. Ed. Engl. 49, 3621–3626. doi: 10.1002/anie.200907210, 20391548

[ref38] Elhassan E. Devnarain N. Mohammed M. Govender T. Omolo C. A. (2022). Engineering hybrid nanosystems for efficient and targeted delivery against bacterial infections. J. Control. Release 351, 598–622. doi: 10.1016/j.jconrel.2022.09.052 36183972

[ref39] Fang J. Nakamura H. Maeda H. (2011). The EPR effect: unique features of tumor blood vessels for drug delivery, factors involved, and limitations and augmentation of the effect. Adv. Drug Deliv. Rev. 63, 136–151. doi: 10.1016/j.addr.2010.04.009, 20441782

[ref40] Farah H. Kadhim-Abosaoda M. Mohaisen-Mousa H. Renuka Jyothi S. Priyadarshini-Nayak P. Bethanney Janney J. . (2025). Nanomedicine strategies against biofilm-associated infections: advances, challenges, and translational barriers. Microbiology 15:e70210. doi: 10.1002/mbo3.70210, 41457060 PMC12745177

[ref41] Faria M. Björnmalm M. Thurecht K. J. Kent S. J. Parton R. G. Kavallaris M. . (2018). Minimum information reporting in bio-nano experimental literature. Nat. Nanotechnol. 13, 777–785. doi: 10.1038/s41565-018-0246-4, 30190620 PMC6150419

[ref42] Filkins L. M. O’Toole G. A. (2015). Cystic fibrosis lung infections: Polymicrobial, complex, and hard to treat. PLoS Pathog. 11:e1005258. doi: 10.1371/journal.ppat.1005258, 26719892 PMC4700991

[ref43] Flannagan R. S. Jaumouillé V. Grinstein S. (2012). The cell biology of phagocytosis. Annu. Rev. Pathol. 7, 61–98. doi: 10.1146/annurev-pathol-011811-132445 21910624

[ref44] Flemming H.-C. Wingender J. (2010). The biofilm matrix. Nat. Rev. Microbiol. 8, 623–633. doi: 10.1038/nrmicro2415, 20676145

[ref45] Flemming H.-C. Wingender J. Szewzyk U. Steinberg P. Rice S. A. Kjelleberg S. (2016). Biofilms: an emergent form of bacterial life. Nat. Rev. Microbiol. 14, 563–575. doi: 10.1038/nrmicro.2016.94 27510863

[ref46] Flores-Vargas G. Korber D. R. Bergsveinson J. (2023). Sub-MIC antibiotics influence the microbiome, resistome and structure of riverine biofilm communities. Front. Microbiol. 14:1194952. doi: 10.3389/fmicb.2023.1194952, 37593545 PMC10427767

[ref47] Gach M. W. Lazarus G. Simadibrata D. M. Sinto R. Saharman Y. R. Limato R. . (2024). Antimicrobial resistance among common bacterial pathogens in Indonesia: a systematic review. The Lancet Regional Health - Southeast Asia 26:100414. doi: 10.1016/j.lansea.2024.100414, 38778837 PMC11109028

[ref48] Gao W. Chan J. Farokhzad O. C. (2010). pH-responsive nanoparticles for drug delivery. Mol. Pharm. 7, 1913–1920. doi: 10.1021/mp100253e, 20836539 PMC3379544

[ref49] Gao F. Xiong Z. (2021). Reactive oxygen species responsive polymers for drug delivery systems. Front. Chem. 9:649048. doi: 10.3389/fchem.2021.649048, 33968898 PMC8103170

[ref50] Germain M. Caputo F. Metcalfe S. Tosi G. Spring K. Åslund A. K. O. . (2020). Delivering the power of nanomedicine to patients today. J. Control. Release 326, 164–171. doi: 10.1016/j.jconrel.2020.07.007, 32681950 PMC7362824

[ref51] Gillies E. R. Fréchet J. M. J. (2005). pH-responsive copolymer assemblies for controlled release of doxorubicin. Bioconjug. Chem. 16, 361–368. doi: 10.1021/bc049851c, 15769090

[ref52] Graham-Gurysh E. G. Murthy A. B. Moore K. M. Hingtgen S. D. Bachelder E. M. Ainslie K. M. (2020). Synergistic drug combinations for a precision medicine approach to interstitial glioblastoma therapy. J. Control. Release 323, 282–292. doi: 10.1016/j.jconrel.2020.04.028, 32335153 PMC7453575

[ref53] Gui S. Li X. Feng M. Liu H. Huang L. Niu X. (2023). A fresh pH-responsive imipenem-loaded nanocarrier against *Acinetobacter baumannii* with a synergetic effect. Front. Bioeng. Biotechnol. 11:1166790. doi: 10.3389/fbioe.2023.1166790, 37113664 PMC10128990

[ref54] Gullberg E. Cao S. Berg O. G. Ilbäck C. Sandegren L. Hughes D. . (2011). Selection of resistant bacteria at very low antibiotic concentrations. PLoS Pathog. 7:e1002158. doi: 10.1371/journal.ppat.1002158, 21811410 PMC3141051

[ref55] Hadianamrei R. Wang J. Brown S. Zhao X. (2022). Rationally designed cationic amphiphilic peptides for selective gene delivery to cancer cells. Int. J. Pharm. 617:121619. doi: 10.1016/j.ijpharm.2022.121619, 35218898

[ref56] Hall C. W. Mah T.-F. (2017). Molecular mechanisms of biofilm-based antibiotic resistance and tolerance in pathogenic bacteria. FEMS Microbiol. Rev. 41, 276–301. doi: 10.1093/femsre/fux010, 28369412

[ref57] Han X. Alu A. Liu H. Shi Y. Wei X. Cai L. . (2022). Biomaterial-assisted biotherapy: a brief review of biomaterials used in drug delivery, vaccine development, gene therapy, and stem cell therapy. Bioact. Mater. 17, 29–48. doi: 10.1016/j.bioactmat.2022.01.011, 35386442 PMC8958282

[ref58] Hare J. I. Lammers T. Ashford M. B. Puri S. Storm G. Barry S. T. (2017). Challenges and strategies in anti-cancer nanomedicine development: an industry perspective. Adv. Drug Deliv. Rev. 108, 25–38. doi: 10.1016/j.addr.2016.04.025, 27137110

[ref59] Hassall J. Coxon C. Patel V. C. Goldenberg S. D. Sergaki C. (2024). Limitations of current techniques in clinical antimicrobial resistance diagnosis: examples and future prospects. NPJ Antimicrob Resist 2:16. doi: 10.1038/s44259-024-00033-8, 39843577 PMC11721362

[ref60] Haval M. Unakal C. Ghagane S. C. Pandit B. R. Daniel E. Siewdass P. . (2025). Biofilms exposed: innovative imaging and therapeutic platforms for persistent infections. Antibiotics 14:865. doi: 10.3390/antibiotics14090865, 41009844 PMC12466655

[ref61] Hindieh P. Yaghi J. Assaf J. C. Chokr A. Atoui A. Tzenios N. . (2025). Emerging multimodal strategies for bacterial biofilm eradication: a comprehensive review. Microorganisms 13:2796. doi: 10.3390/microorganisms13122796, 41471999 PMC12735920

[ref62] Hofer U. (2021). Tracking down polyclonal tuberculosis. Nat. Rev. Microbiol. 19:406. doi: 10.1038/s41579-021-00580-1, 34017088

[ref63] Hosseini S. M. Taheri M. Nouri F. Farmani A. Moez N. M. Arabestani M. R. (2022). Nano drug delivery in intracellular bacterial infection treatments. Biomed. Pharmacother. 146:112609. doi: 10.1016/j.biopha.2021.112609 35062073

[ref64] Hu Q. Katti P. S. Gu Z. (2014). Enzyme-responsive nanomaterials for controlled drug delivery. Nanoscale 6, 12273–12286. doi: 10.1039/c4nr04249b, 25251024 PMC4425417

[ref65] Huang X. Brazel C. S. (2001). On the importance and mechanisms of burst release in matrix-controlled drug delivery systems. J. Control. Release 73, 121–136. doi: 10.1016/s0168-3659(01)00248-6, 11516493

[ref66] Hunter C. A. Jones S. A. (2015). IL-6 as a keystone cytokine in health and disease. Nat. Immunol. 16, 448–457. doi: 10.1038/ni.3153, 25898198

[ref67] Jacob S. Rao R. Gorain B. Boddu S. H. S. Nair A. B. (2025). Solid lipid nanoparticles and nanostructured lipid carriers for anticancer phytochemical delivery: advances, challenges, and future prospects. Pharmaceutics 17:1079. doi: 10.3390/pharmaceutics17081079, 40871098 PMC12389418

[ref68] Jiang J.-H. Cameron D. R. Nethercott C. Aires-de-Sousa M. Peleg A. Y. (2023). Virulence attributes of successful methicillin-resistant *Staphylococcus aureus* lineages. Clin. Microbiol. Rev. 36:e00148-22. doi: 10.1128/cmr.00148-22, 37982596 PMC10732075

[ref69] Jumadi J. Harun W. S. W. Kadirgama K. Samylingam L. Aslfattahi N. Clint K. S. . (2025). A comparative review of polylactic acid and poly(lactic-co-glycolic acid) biomaterials: optimizing drug delivery. BioNanoSci 16:19. doi: 10.1007/s12668-025-02233-6

[ref70] Kamaly N. Yameen B. Wu J. Farokhzad O. C. (2016). Degradable controlled-release polymers and polymeric nanoparticles: mechanisms of controlling drug release. Chem. Rev. 116, 2602–2663. doi: 10.1021/acs.chemrev.5b00346, 26854975 PMC5509216

[ref71] Karaiskos I. Giamarellou H. (2014). Multidrug-resistant and extensively drug-resistant gram-negative pathogens: current and emerging therapeutic approaches. Expert. Opin. Pharmacother. 15, 1351–1370. doi: 10.1517/14656566.2014.914172, 24766095 PMC4819585

[ref72] Karimi M. Eslami M. Sahandi-Zangabad P. Mirab F. Farajisafiloo N. Shafaei Z. . (2016). pH-sensitive stimulus-responsive nanocarriers for targeted delivery of therapeutic agents. Wiley Interdiscip. Rev. Nanomed. Nanobiotechnol. 8, 696–716. doi: 10.1002/wnan.1389, 26762467 PMC4945487

[ref73] Karimlar S. Pirbalouti A. G. Teymuori Z. Moslehishad M. Hamidi-Esfahani Z. (2026). Applications of chitosan, an eco-friendly biopolymer in agricultural systems, herbal products, and functional foods: a review. Food Sci. Nutr. 14:e71367. doi: 10.1002/fsn3.71367, 41497730 PMC12765821

[ref74] Kolge H. Ahmad Z. Singh S. Yu M. Kumar A. (2026). Chitosan-coated silver–vancomycin nanoparticles for treatment of bacterial endophthalmitis. Invest. Ophthalmol. Vis. Sci. 67:20. doi: 10.1167/iovs.67.3.20, 41805149 PMC12988689

[ref75] Kumar M. Hilles A. R. Almurisi S. H. Bhatia A. Mahmood S. (2023). Micro and nano-carriers-based pulmonary drug delivery system: their current updates, challenges, and limitations – a review. JCIS Open 12:100095. doi: 10.1016/j.jciso.2023.100095

[ref76] Kunjachan S. Rychlik B. Storm G. Kiessling F. Lammers T. (2013). Multidrug resistance: physiological principles and nanomedical solutions. Adv. Drug Deliv. Rev. 65, 1852–1865. doi: 10.1016/j.addr.2013.09.018, 24120954 PMC3939439

[ref77] Lebeaux D. Ghigo J.-M. Beloin C. (2014). Biofilm-related infections: bridging the gap between clinical management and fundamental aspects of recalcitrance toward antibiotics. Microbiol. Mol. Biol. Rev. 78, 510–543. doi: 10.1128/MMBR.00013-14, 25184564 PMC4187679

[ref78] Lee Y. Thompson D. H. (2017). Stimuli-responsive liposomes for drug delivery. WIREs Nanomed. Nanobiotechnol. 9:10.1002/wnan.1450. doi: 10.1002/wnan.1450, 28198148 PMC5557698

[ref79] Lee H.-J. Woo Y. Hahn T.-W. Jung Y. M. Jung Y.-J. (2020). Formation and maturation of the phagosome: a key mechanism in innate immunity against intracellular bacterial infection. Microorganisms 8:1298. doi: 10.3390/microorganisms8091298, 32854338 PMC7564318

[ref80] Lewis K. (2010). Persister cells. Ann. Rev. Microbiol. 64, 357–372. doi: 10.1146/annurev.micro.112408.134306, 20528688

[ref81] Li P. Pan J. Dong Y. Sun Y. Wang Y. Liao K. . (2024). Microenvironment responsive charge-switchable nanoparticles act on biofilm eradication and virulence inhibition for chronic lung infection treatment. J. Control. Release 365, 219–235. doi: 10.1016/j.jconrel.2023.11.032, 37992874

[ref82] Li X.-Z. Plésiat P. Nikaido H. (2015). The challenge of efflux-mediated antibiotic resistance in gram-negative Bacteria. Clin. Microbiol. Rev. 28, 337–418. doi: 10.1128/CMR.00117-14, 25788514 PMC4402952

[ref83] Liang J. Liu B. (2016). ROS-responsive drug delivery systems. Bioeng. Transl. Med. 1, 239–251. doi: 10.1002/btm2.10014, 29313015 PMC5689534

[ref84] Lister J. L. Horswill A. R. (2014). *Staphylococcus aureus* biofilms: recent developments in biofilm dispersal. Front. Cell. Infect. Microbiol. 4:178. doi: 10.3389/fcimb.2014.00178, 25566513 PMC4275032

[ref85] Liu H. Y. Prentice E. L. Webber M. A. (2024a). Mechanisms of antimicrobial resistance in biofilms. NPJ Antimicrob Resist 2:27. doi: 10.1038/s44259-024-00046-3, 39364333 PMC11445061

[ref86] Liu Y. Wang W. Yang J. Zhou C. Sun J. (2013). pH-sensitive polymeric micelles triggered drug release for extracellular and intracellular drug targeting delivery. Asian J. Pharm. Sci. 8, 159–167. doi: 10.1016/j.ajps.2013.07.021

[ref87] Liu Y. Wei Z. Mutti F. F. Zhang H. Loeffler F. F. (2024b). Redox-responsive inorganic fluorescent nanoprobes for serodiagnosis and bioimaging. Coord. Chem. Rev. 509:215817. doi: 10.1016/j.ccr.2024.215817

[ref88] Lu Y. Shan P. Lu W. Yin X. Liu H. Lian X. . (2023a). ROS-responsive and self-amplifying polymeric prodrug for accelerating infected wound healing. Chem. Eng. J. 463:142311. doi: 10.1016/j.cej.2023.142311

[ref89] Lu Y. Shi Y. Wu Q. Sun X. Zhang W.-Z. Xu X.-L. . (2023b). An overview of drug delivery Nanosystems for Sepsis-related liver injury treatment. Int. J. Nanomedicine 18, 765–779. doi: 10.2147/IJN.S394802, 36820059 PMC9938667

[ref90] Lyon P. C. Gray M. D. Mannaris C. Folkes L. K. Stratford M. Campo L. . (2018). Safety and feasibility of ultrasound-triggered targeted drug delivery of doxorubicin from thermosensitive liposomes in liver tumours (TARDOX): a single-Centre, open-label, phase 1 trial. Lancet Oncol. 19, 1027–1039. doi: 10.1016/S1470-2045(18)30332-2, 30001990 PMC6073884

[ref91] Maeda H. Tsukigawa K. Fang J. (2016). A retrospective 30 years after discovery of the enhanced permeability and retention effect of solid tumors: next-generation chemotherapeutics and photodynamic therapy--problems, solutions, and prospects. Microcirculation 23, 173–182. doi: 10.1111/micc.12228, 26237291

[ref92] Malainou C. Abdin S. M. Lachmann N. Matt U. Herold S. (2023). Alveolar macrophages in tissue homeostasis, inflammation, and infection: evolving concepts of therapeutic targeting. J. Clin. Invest. 133:e170501. doi: 10.1172/JCI170501, 37781922 PMC10541196

[ref93] Mamnoon B. Feng L. Froberg J. Choi Y. Venkatachalem S. Mallik S. (2020). Hypoxia-responsive, polymeric Nanocarriers for targeted drug delivery to estrogen receptor-positive breast Cancer cell spheroids. Mol. Pharm. 17, 4312–4322. doi: 10.1021/acs.molpharmaceut.0c00754, 32926627 PMC8095663

[ref94] Mehta M. Bui T. A. Yang X. Aksoy Y. Goldys E. M. Deng W. (2023). Lipid-based nanoparticles for drug/gene delivery: an overview of the production techniques and difficulties encountered in their industrial development. ACS Mater Au 3, 600–619. doi: 10.1021/acsmaterialsau.3c00032, 38089666 PMC10636777

[ref95] Mi P. (2020). Stimuli-responsive nanocarriers for drug delivery, tumor imaging, therapy and theranostics. Theranostics 10, 4557–4588. doi: 10.7150/thno.38069, 32292515 PMC7150471

[ref96] Moghimi S. M. Andersen A. J. Ahmadvand D. Wibroe P. P. Andresen T. L. Hunter A. C. (2011). Material properties in complement activation. Adv. Drug Deliv. Rev. 63, 1000–1007. doi: 10.1016/j.addr.2011.06.002, 21689701

[ref97] Morales D. R. Slattery J. Pacurariu A. Pinheiro L. McGettigan P. Kurz X. (2019). Relative and absolute risk of tendon rupture with fluoroquinolone and concomitant fluoroquinolone/corticosteroid therapy: population-based nested case-control study. Clin. Drug Investig. 39, 205–213. doi: 10.1007/s40261-018-0729-y, 30465300 PMC6394638

[ref98] Mora-Ochomogo M. Lohans C. T. (2021). β-Lactam antibiotic targets and resistance mechanisms: from covalent inhibitors to substrates. RSC Med. Chem. 12, 1623–1639. doi: 10.1039/d1md00200g, 34778765 PMC8528271

[ref99] Mukhopadhyay H. Bairagi A. Mukherjee A. Prasad A. K. Roy A. D. Nayak A. (2025). Multidrug resistant *Acinetobacter baumannii*: a study on its pathogenesis and therapeutics. Curr. Res. Microb. Sci. 8:100331. doi: 10.1016/j.crmicr.2024.100331, 39802320 PMC11718326

[ref100] Mura S. Nicolas J. Couvreur P. (2013). Stimuli-responsive nanocarriers for drug delivery. Nat. Mater. 12, 991–1003. doi: 10.1038/nmat3776, 24150417

[ref101] Murray C. J. L. Ikuta K. S. Sharara F. Swetschinski L. Aguilar G. R. Gray A. . (2022). Global burden of bacterial antimicrobial resistance in 2019: a systematic analysis. Lancet 399, 629–655. doi: 10.1016/S0140-6736(21)02724-0, 35065702 PMC8841637

[ref102] Naseri N. Valizadeh H. Zakeri-Milani P. (2015). Solid lipid nanoparticles and nanostructured lipid carriers: structure, preparation and application. Adv Pharm Bull 5, 305–313. doi: 10.15171/apb.2015.043, 26504751 PMC4616893

[ref103] Nathan C. Cunningham-Bussel A. (2013). Beyond oxidative stress: an immunologist’s guide to reactive oxygen species. Nat. Rev. Immunol. 13, 349–361. doi: 10.1038/nri3423, 23618831 PMC4250048

[ref104] Nekoofar M. H. Namazikhah M. S. Sheykhrezae M. S. Mohammadi M. M. Kazemi A. Aseeley Z. . (2009). pH of pus collected from periapical abscesses. Int. Endod. J. 42, 534–538. doi: 10.1111/j.1365-2591.2009.01550.x, 19460003

[ref105] Nkune N. W. Abrahamse H. (2025). Novel nanoplatforms for antimicrobial photodynamic inactivation of bacterial biofilm infections. Photodiagn. Photodyn. Ther. 56:105297. doi: 10.1016/j.pdpdt.2025.105297, 41276197

[ref106] Nnenna Ugwu C. Nneoma Ezeibe E. Chijioke Emencheta S. Sherridan Nwagwu C. Onyenonachi Ogbonna K. Victor Ejiofor C. . (2025). Biofilms: structure, resistance mechanism, emerging control strategies, and applications. RSC Pharmaceutics 2, 1376–1407. doi: 10.1039/D5PM00094G

[ref107] Olivier K. N. Griffith D. E. Eagle G. McGinnis J. P. Micioni L. Liu K. . (2017). Randomized trial of liposomal amikacin for inhalation in nontuberculous mycobacterial lung disease. Am. J. Respir. Crit. Care Med. 195, 814–823. doi: 10.1164/rccm.201604-0700OC, 27748623 PMC5363966

[ref108] Pandey V. Pandey T. (2025). A mechanistic understanding of reactive oxygen species (ROS)-responsive bio-polymeric nanoparticles: current state, challenges and future toward precision therapeutics. Biopolymers 116:e70027. doi: 10.1002/bip.70027, 40370134

[ref109] Pearson R. M. Casey L. M. Hughes K. R. Miller S. D. Shea L. D. (2017). *In vivo* reprogramming of immune cells: technologies for induction of antigen-specific tolerance. Adv. Drug Deliv. Rev. 114, 240–255. doi: 10.1016/j.addr.2017.04.005, 28414079 PMC5582017

[ref110] Pelgrift R. Y. Friedman A. J. (2013). Nanotechnology as a therapeutic tool to combat microbial resistance. Adv. Drug Deliv. Rev. 65, 1803–1815. doi: 10.1016/j.addr.2013.07.011, 23892192

[ref111] Peng S. Xiao F. Chen M. Gao H. (2021). Tumor-microenvironment-responsive nanomedicine for enhanced cancer immunotherapy. Adv. Sci. 9:2103836. doi: 10.1002/advs.202103836, 34796689 PMC8728817

[ref112] Percival S. L. McCarty S. Hunt J. A. Woods E. J. (2014). The effects of pH on wound healing, biofilms, and antimicrobial efficacy. Wound Repair Regen. 22, 174–186. doi: 10.1111/wrr.12125 24611980

[ref113] Perrigue P. M. Murray R. A. Mielcarek A. Henschke A. Moya S. E. (2021). Degradation of drug delivery Nanocarriers and payload release: a review of physical methods for tracing Nanocarrier biological fate. Pharmaceutics 13:770. doi: 10.3390/pharmaceutics13060770, 34064155 PMC8224277

[ref114] Price S. L. Skaar E. P. (2025). Forging biofilms: metal-induced microbial responses in biofilm formation. J. Bacteriol. 207:e00247-25. doi: 10.1128/jb.00247-25, 41099530 PMC12632276

[ref115] Rajkhowa S. Hussain S. Z. Agarwal M. Zaheen A. Al-Hussain S. A. Zaki M. E. A. (2024). Advancing antibiotic-resistant microbe combat: Nanocarrier-based Systems in Combination Therapy Targeting Quorum Sensing. Pharmaceutics 16:1160. doi: 10.3390/pharmaceutics16091160, 39339197 PMC11434747

[ref116] Rinaldi A. Caraffi R. Grazioli M. V. Oddone N. Giardino L. Tosi G. . (2022). Applications of the ROS-responsive Thioketal linker for the production of smart nanomedicines. Polymers 14:687. doi: 10.3390/polym14040687, 35215600 PMC8874672

[ref117] Schulte W. Bernhagen J. Bucala R. (2013). Cytokines in sepsis: potent immunoregulators and potential therapeutic targets--an updated view. Mediat. Inflamm. 2013:165974. doi: 10.1155/2013/165974, 23853427 PMC3703895

[ref118] Seebach E. Kubatzky K. F. (2019). Chronic implant-related bone infections—can immune modulation be a therapeutic strategy? Front. Immunol. 10:1724. doi: 10.3389/fimmu.2019.01724, 31396229 PMC6664079

[ref119] Segal A. W. (2008). The function of the NADPH oxidase of phagocytes and its relationship to other NOXs in plants, invertebrates, and mammals. Int. J. Biochem. Cell Biol. 40, 604–618. doi: 10.1016/j.biocel.2007.10.003, 18036868 PMC2636181

[ref120] Sharif S. Yadav A. K. (2025). Bacterial biofilm and its role in antibiotic resistance. Microbe 7:100356. doi: 10.1016/j.microb.2025.100356

[ref121] Sobczak M. (2022). Enzyme-responsive hydrogels as potential drug delivery systems—state of knowledge and future prospects. Int. J. Mol. Sci. 23:4421. doi: 10.3390/ijms23084421, 35457239 PMC9031066

[ref122] Stone N. R. H. Bicanic T. Salim R. Hope W. (2016). Liposomal amphotericin B (AmBisome(®)): a review of the pharmacokinetics, pharmacodynamics, clinical experience and future directions. Drugs 76, 485–500. doi: 10.1007/s40265-016-0538-7, 26818726 PMC4856207

[ref123] Sun Z. Xiao M. Lv S. Wang C. Fu H. Tian L. . (2025). A pH-responsive, surface charge-switchable nanosystem with enhanced biofilm penetration for synergistic photodynamic and antibiotic therapy of diabetic wounds. Adv. Funct. Mater. 35:2418711. doi: 10.1002/adfm.202418711

[ref125] Tang R. Tan H. Dai Y. Li L. Huang Y. Yao H. . (2023). Application of antimicrobial peptides in plant protection: making use of the overlooked merits. Front. Plant Sci. 14:1139539. doi: 10.3389/fpls.2023.1139539, 37538059 PMC10394246

[ref126] Thakur A. Mikkelsen H. Jungersen G. (2019). Intracellular pathogens: host immunity and microbial persistence strategies. J. Immunol. Res. 2019, 1–24. doi: 10.1155/2019/1356540, 31111075 PMC6487120

[ref127] Tooke C. L. Hinchliffe P. Bragginton E. C. Colenso C. K. Hirvonen V. H. A. Takebayashi Y. . (2019). β-Lactamases and β-lactamase inhibitors in the 21st century. J. Mol. Biol. 431, 3472–3500. doi: 10.1016/j.jmb.2019.04.002, 30959050 PMC6723624

[ref128] Torchilin V. P. (2014). Multifunctional, stimuli-sensitive nanoparticulate systems for drug delivery. Nat. Rev. Drug Discov. 13, 813–827. doi: 10.1038/nrd4333, 25287120 PMC4489143

[ref129] Torres-Herrero B. Armenia I. Ortiz C. de la Fuente J. M. Betancor L. Grazú V. (2024). Opportunities for nanomaterials in enzyme therapy. J. Control. Release 372, 619–647. doi: 10.1016/j.jconrel.2024.06.035, 38909702

[ref130] Tracey K. J. Cerami A. (1994). Tumor necrosis factor: a pleiotropic cytokine and therapeutic target. Annu. Rev. Med. 45, 491–503. doi: 10.1146/annurev.med.45.1.491, 8198398

[ref131] Ulhuq F. R. Mariano G. (2022). Bacterial pore-forming toxins. Microbiology 168:001154. doi: 10.1099/mic.0.001154, 35333704 PMC9558359

[ref132] Ventola C. L. (2017). Progress in nanomedicine: approved and investigational Nanodrugs. P T 42, 742–755, 29234213 PMC5720487

[ref133] Winterbourn C. C. Hampton M. B. (2015). Redox biology: signaling via a peroxiredoxin sensor. Nat. Chem. Biol. 11, 5–6. doi: 10.1038/nchembio.1722, 25517384

[ref134] Xia Z. Mu W. Yuan S. Fu S. Liu Y. Zhang N. (2023). Cell membrane biomimetic Nano-delivery Systems for Cancer Therapy. Pharmaceutics 15:2770. doi: 10.3390/pharmaceutics15122770, 38140108 PMC10748133

[ref135] Xiong M.-H. Li Y.-J. Bao Y. Yang X.-Z. Hu B. Wang J. (2012). Bacteria-responsive multifunctional nanogel for targeted antibiotic delivery. Adv. Mater. 24, 6175–6180. doi: 10.1002/adma.201202847, 22961974

[ref136] Zhang L. Tian X. Sun L. Mi K. Wang R. Gong F. . (2024). Bacterial efflux pump inhibitors reduce antibiotic resistance. Pharmaceutics 16:170. doi: 10.3390/pharmaceutics16020170, 38399231 PMC10892612

[ref137] Zheng Z. Cui J. Wu S. Cao Z. Cao P. (2025). Engineering metal-organic frameworks for enhanced antimicrobial efficacy: synthesis methodologies, mechanistic perspectives, and versatile applications. J. Funct. Biomater. 16:353. doi: 10.3390/jfb16090353, 41003425 PMC12470498

[ref138] Zhou Q. Si Z. Wang K. Li K. Hong W. Zhang Y. . (2022). Enzyme-triggered smart antimicrobial drug release systems against bacterial infections. J. Control. Release 352, 507–526. doi: 10.1016/j.jconrel.2022.10.038, 36341932

[ref139] Zhou Q. Zhang L. Yang T. Wu H. (2018). Stimuli-responsive polymeric micelles for drug delivery and cancer therapy. Int. J. Nanomedicine 13, 2921–2942. doi: 10.2147/IJN.S158696, 29849457 PMC5965378

